# EZH2 inhibition reactivates epigenetically silenced *FMR1* and normalizes molecular and electrophysiological abnormalities in fragile X syndrome neurons

**DOI:** 10.3389/fnins.2024.1348478

**Published:** 2024-02-21

**Authors:** Minggang Fang, Sara K. Deibler, Pranathi Meda Krishnamurthy, Feng Wang, Paola Rodriguez, Shahid Banday, Ching-Man Virbasius, Miguel Sena-Esteves, Jonathan K. Watts, Michael R. Green

**Affiliations:** ^1^Department of Molecular, Cell and Cancer Biology, University of Massachusetts Chan Medical School, Worcester, MA, United States; ^2^RNA Therapeutics Institute, University of Massachusetts Chan Medical School, Worcester, MA, United States; ^3^Department of Neurology, University of Massachusetts Chan Medical School, Worcester, MA, United States

**Keywords:** FXS, *FMR1* silencing, *FMR1* reactivation, EZH2 (enhancer of zeste homolog 2), ASO - antisense oligonucleotides, neuron differentiation

## Abstract

Fragile X Syndrome (FXS) is a neurological disorder caused by epigenetic silencing of the *FMR1* gene. Reactivation of *FMR1* is a potential therapeutic approach for FXS that would correct the root cause of the disease. Here, using a candidate-based shRNA screen, we identify nine epigenetic repressors that promote silencing of *FMR1* in FXS cells (called *FMR1* Silencing Factors, or *FMR1*- SFs). Inhibition of *FMR1*-SFs with shRNAs or small molecules reactivates *FMR1* in cultured undifferentiated induced pluripotent stem cells, neural progenitor cells (NPCs) and post-mitotic neurons derived from FXS patients. One of the *FMR1*-SFs is the histone methyltransferase EZH2, for which an FDA-approved small molecule inhibitor, EPZ6438 (also known as tazemetostat), is available. We show that EPZ6438 substantially corrects the characteristic molecular and electrophysiological abnormalities of cultured FXS neurons. Unfortunately, EZH2 inhibitors do not efficiently cross the blood-brain barrier, limiting their therapeutic use for FXS. Recently, antisense oligonucleotide (ASO)-based approaches have been developed as effective treatment options for certain central nervous system disorders. We therefore derived efficacious ASOs targeting EZH2 and demonstrate that they reactivate *FMR1* expression and correct molecular and electrophysiological abnormalities in cultured FXS neurons, and reactivate *FMR1* expression in human FXS NPCs engrafted within the brains of mice. Collectively, our results establish EZH2 inhibition in general, and EZH2 ASOs in particular, as a therapeutic approach for FXS.

## Summary

EZH2 inhibition normalizes cultured Fragile X Syndrome neurons and reactivates *hFMR1* in mice, suggesting a new therapeutic approach for the disease.

## Introduction

Fragile X Syndrome (FXS) is the most common inherited form of intellectual disability and most prevalent monogenic cause of autism, occurring in ∼1 in 4,000 males and ∼1 in 8,000 females (Tassanakijpanich et al., 2021). Currently there are no curative therapies for FXS. The disease is caused by a CGG repeat expansion in the 5′ untranslated region of the X-linked *FMR1* gene. Normal individuals have 6–54 repeats, whereas expansion of the repeats to >200 results in an *FMR1* full mutation, which leads to transcriptional inactivation of *FMR1* by a process referred to as epigenetic silencing. The epigenetically silenced *FMR1* gene has the typical hallmarks of heterochromatin including DNA hypermethylation, gain of repressive histone modifications such as H3 lysine 9 trimethylation (H3K9me3), H3 lysine 27 trimethylation (H3K27me3) and H4 lysine 20 trimethylation (H4K20me3), and loss of activating histone modifications such as H3 lysine 4 trimethylation (H3K4me3) and H2A/H2B/H3/H4 acetylation (Oberle et al., 1991; Coffee et al., 1999, 2002; Pietrobono et al., 2005; Tabolacci et al., 2005, 2008a; Kumari and Usdin, 2010). Although the epigenetic marks on the silenced *FMR1* promoter are known, the specific factors that write, read or erase these marks are surprisingly poorly understood.

As a consequence of *FMR1* silencing, the product of *FMR1*, the fragile X mental retardation protein (FMRP), is not produced. FMRP is a highly conserved protein expressed in all cells but is particularly prevalent in the brain (Verkerk et al., 1991; Devys et al., 1993; Santoro et al., 2012). FMRP is an RNA-binding protein that predominantly functions by repressing mRNA translation, and in its absence protein synthesis is excessive, which results in disease pathology (Richter et al., 2015). In FXS neurons, the translation dysfunction results in several characteristic molecular abnormalities including increased levels of the neuronal-specific transcription repressor REST (Halevy et al., 2015), decreased expression of axonal guidance genes (Halevy et al., 2015), and diminished levels of the signaling protein diacylglycerol kinase kappa (DGKK) (Tabet et al., 2016). FMRP has also been proposed to have other activities, including direct regulation of ion channels (Deng et al., 2013).

A prominent manifestation of FXS is synaptic weakening, which is measured electrophysiologically as long-term depression (LTD). A particular form of LTD, metabotropic glutamate receptor 5 (mGluR5)-LTD, is abnormally exaggerated in FXS, which has given rise to the so-called mGluR theory of FXS (Bear et al., 2004). This theory posits that inhibition of mGluR5 signaling should reverse or rescue pathophysiologies associated with the disease. In support of the mGluR theory, inhibition of mGluR5 signaling corrects disease symptomatology in *FMR1*^–/–^ knockout mice, the major animal model of FXS (Dolen et al., 2007). However, large- scale clinical trials using mGluR5 antagonists have shown no beneficial effect in FXS patients (Berry-Kravis et al., 2016; Youssef et al., 2018). Thus, there is a great need for therapeutic approaches that are not based on mGluR5 inhibition.

Reactivation of the epigenetically silenced *FMR1* gene is a potential therapeutic approach for FXS that would correct the root cause of the disease, the aberrant gene expression, rather than a secondary, downstream consequence of FMRP deficiency, such as increased mGluR5 signaling (Shitik et al., 2020). Several considerations suggest that reactivation of epigenetically silenced *FMR1* in FXS patients will result in increased expression of *FMR1* and decreased disease pathology. First, targeted deletion (Park et al., 2015; Xie et al., 2016) or demethylation (Liu et al., 2018) of the *FMR1* CGG repeats in cultured FXS cells results in transcription reactivation of *FMR1*. Second, proof-of-principle studies in mouse and *Drosophila* models of FXS have shown that disease symptoms are reversible (McBride et al., 2005; Zeier et al., 2009; Henderson et al., 2012; Gholizadeh et al., 2014; Gkogkas et al., 2014). Finally, there are rare asymptomatic individuals who have an *FMR1* full mutation but still express *FMR1* (Tabolacci et al., 2008b), suggesting that restoration of *FMR1* transcription in an FXS mutant background will ameliorate disease.

To further explore the feasibility of the *FMR1* reactivation approach, here we perform a candidate-based RNA interference (RNAi) screen to identify epigenetic regulators that promote silencing of *FMR1* in FXS cells. Using that information, we then find nucleic acid-based and small molecule inhibitors of the factors that promote epigenetic silencing, and show that they reactivate the epigenetically silenced *FMR1* gene in cultured FXS cells. Finally, we show that antisense oligonucleotide (ASO)-mediated inhibition of one of the factors, the H3K27 methyltransferase EZH2, reactivates *FMR1* expression and substantially corrects characteristic molecular and electrophysiological abnormalities in cultured FXS neurons, and reactivates *FMR1* in human FXS neural progenitor cells engrafted within the brains of mice. Our results establish the feasibility of *FMR1* reactivation as a therapeutic approach for FXS.

## Results

### A candidate-based RNAi screen identifies epigenetic regulators that mediate silencing of *FMR1* in FXS patient-derived induced pluripotent stem cells

**FIGURE 1:**
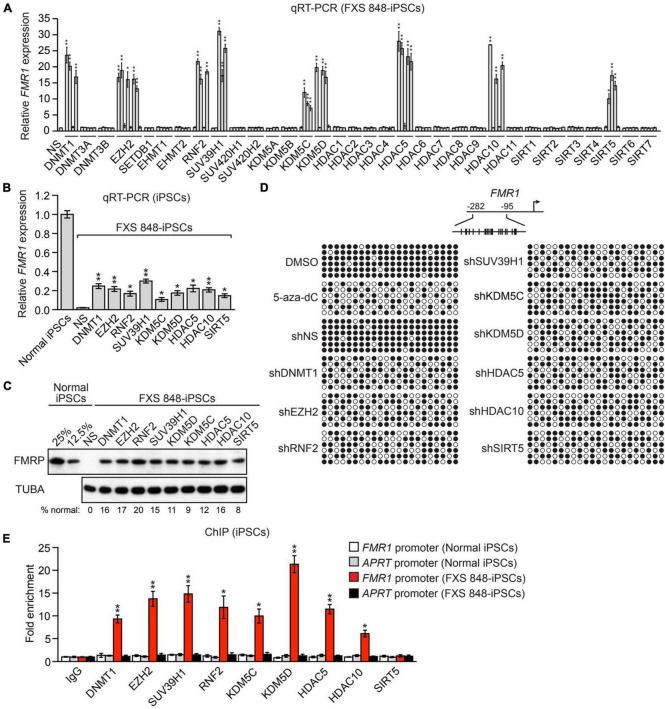
A candidate-based RNAi screen identifies epigenetic regulators that mediate silencing of *FMR1* in FXS patient-derived iPSCs. **(A)** qRT-PCR analysis monitoring expression of *FMR1* in FXS 848-iPSCs expressing one of 162 shRNAs targeting a set of 33 epigenetic repressors. The results were normalized to that obtained with a control non-silencing (NS) shRNA, which was set to 1. **(B)** qRT-PCR analysis monitoring *FMR1* expression in FXS 848-iPSCs 20 days following expression of an *FMR1*-SF shRNA. The results were normalized to that obtained in normal iPSCs, which was set to 1. **(C)** Immunoblot analysis showing FMRP protein levels in FXS 848-iPSCs 20 days following expression of an *FMR1*-SF shRNA. The levels of FMRP in normal iPSCs, diluted fourfold (representing the level of FMRP at 25% of normal levels) and eightfold (12.5%), are shown. α-tubulin (TUBA) was monitored as a loading control. The FMRP signal was quantified and normalized to that obtained in normal iPSCs, which was multiplied by the dilution factor and then set to 100%. **(D)** Bisulfite sequencing analysis of the *FMR1* promoter in FXS 848-iPSCs treated with DMSO or 5-aza-2′-deoxycytidine (5-aza-dC), or with an NS or *FMR1*-SF shRNA. (Top) Schematic of the *FMR1* promoter; positions of CpGs are shown to scale by vertical lines. (Bottom) Each circle represents a methylated (black) or unmethylated (white) CpG dinucleotide. Each row represents a single clone. **(E)** ChIP analysis monitoring binding of *FMR1*-SFs to the *FMR1* promoter in normal and FXS 848-iPSCs. As a negative control, binding was also monitored at the constitutively expressed *APRT* promoter in normal and FXS 848-iPSCs. The results were normalized to that obtained with IgG, which was set to 1. Data are represented as mean ± SD (*n* = 3 biological replicates). *P < 0.05, **P < 0.01.

As a first step toward investigating *FMR1* reactivation as a therapeutic approach for FXS, we sought to identify epigenetic regulators that promote silencing of *FMR1*. Toward this end we assembled a small-scale short hairpin RNA (shRNA) library comprising 162 shRNAs directed against 33 well characterized epigenetic regulators that mediate transcriptional repression and that write or erase the epigenetic marks known to be associated with the epigenetically silenced *FMR1* promoter (Supplementary Figure 1A). Each shRNA was packaged into lentivirus particles and transduced into an induced pluripotent stem cell (iPSC) line derived from a male patient with FXS (FXS 848-iPS3 cells, hereafter called FXS 848-iPSCs) (Sheridan et al., 2011). Twenty days post-transfection, mRNA was prepared and *FMR1* expression analyzed by quantitative RT-PCR (qRT-PCR). We considered a positive result to be at least two unrelated shRNAs directed against the same gene that elicited a statistically significant: (1) increase in *FMR1* expression, and (2) decrease in mRNA levels of the target gene, compared to that obtained with a control non-silencing (NS) shRNA. The results obtained in the complete screen of the 162 shRNAs are shown in Figure 1A (and see also Supplementary Figure 1B) and enabled us to identify nine epigenetic regulators of the silenced *FMR1* gene: DNMT1, EZH2, RNF2 (also called RING1B), SUV39H1, KDM5C, KDM5D, HDAC5, HDAC10, and SIRT5, whose functions are summarized in Supplementary Figure 1C. For convenience, we refer to the factors that promote *FMR1* silencing as *FMR1* Silencing Factors (*FMR1*-SFs). Notably, three of the *FMR1*- SFs we identified, DNMT1, SUV39H1, and EZH2 have been previously implicated in silencing of the CGG repeat-containing *FMR1* gene (Bar-Nur et al., 2012; Bulut-Karslioglu et al., 2012; Kumari and Usdin, 2016; Tabolacci et al., 2016; Kumari et al., 2020; Vershkov et al., 2022).

KDM5C and KDM5D are lysine-specific histone demethylases with 86% amino acid identity and 91% amino acid similarity (Kent-First et al., 1996). It was therefore important to confirm the specificity of the *KDM5C* and *KDM5D* shRNAs. Analyses by qRT-PCR (Supplementary Figure 2A) and immunoblot (Supplementary Figure 2B) show that the *KDM5C* shRNAs efficiently knocked down KDM5C but not KDM5D, whereas the *KDM5D* shRNAs efficiently knocked down KDM5D but not KDM5C. These results confirm the specificity of the KDM5C and KDM5D shRNAs, and in conjunction with our other results presented above and below demonstrate that both KDM5C and KDM5D contribute to epigenetic silencing of *FMR1*.

To determine the level of *FMR1* reactivation obtained following shRNA-mediated knockdown of an *FMR1*-SF we analyzed in parallel an iPSC line derived from a normal individual (BJ1-iPS4 cells), hereafter called normal iPSCs (Sheridan et al., 2011). The qRT-PCR results of Figure 1B show that shRNA-mediated knockdown of an *FMR1*-SF in FXS 848-iPSCs reactivated the epigenetically silenced *FMR1* gene to ∼10–20% of normal levels at 20 days following lentivirus transduction, the time at which we found *FMR1* reactivation was maximal (Supplementary Figure 3A). We observed a similar level of *FMR1* reactivation following knockdown of an *FMR1*-SF in a TaqMan assay (Supplementary Figure 3B). The immunoblot results of Figure 1C show that knockdown of an *FMR1*-SF also restored FMRP protein to ∼10–20% of normal levels. Reactivation of epigenetically silenced *FMR1* following knockdown of each *FMR1*-SF was confirmed by qRT-PCR and immunoblotting in a second male FXS iPSC cell line, SC135 cells [FXS SC135-iPSCs; (Brick et al., 2014; Supplementary Figures 4A–D)]. By contrast, in normal iPSCs, *FMR1*-SF knockdown had no effect on *FMR1* expression (Supplementary Figure 4E).

A characteristic feature of epigenetically silenced *FMR1* is the presence of DNA hypermethylation (Oberle et al., 1991; Pietrobono et al., 2005; Tabolacci et al., 2008a). The bisulfite sequencing experiment of Figure 1D shows, as expected, that the *FMR1* promoter is hypermethylated in FXS 848-iPSCs. Consistent with previous studies (Bar-Nur et al., 2012; Kumari and Usdin, 2014; Tabolacci et al., 2016), treatment of FXS 848-iPSCs with the DNMT inhibitor 5-aza-2′-deoxycytidine (5-aza-dC) led to a substantial decrease in DNA hypermethylation (Figure 1D and Supplementary Figure 5A). Notably, there was a similar decrease in DNA hypermethylation following knockdown of each of the nine *FMR1*-SFs (Figure 1D and Supplementary Figure 5B). Collectively, these results indicate that the nine FMR-SFs we identified mediate silencing of *FMR1* in FXS iPSCs.

Epigenetic regulators are typically stably (but reversibly) associated with the promoters and/or genes upon which they act. To determine whether the nine *FMR1*-SFs are stably associated with the epigenetically silenced *FMR1* promoter we performed a chromatin immunoprecipitation (ChIP) experiments. The ChIP experiment of Figure 1E shows that eight of the nine *FMR1*-SFs are specifically bound to the epigenetically silenced *FMR1* promoter in FXS 848-iPSCs and not the transcriptionally active *FMR1* promoter in normal iPSCs. The single *FMR1*-SF that is not associated with the epigenetically silenced *FMR1* promoter is SIRT5, which is a mitochondrial protein (Michishita et al., 2005). Thus, although SIRT5 promotes *FMR1* silencing, unlike the other *FMR1*-SFs it functions indirectly.

### Reactivation of epigenetically silenced *FMR1* by small molecule *FMR1*-SF inhibitors

**FIGURE 2:**
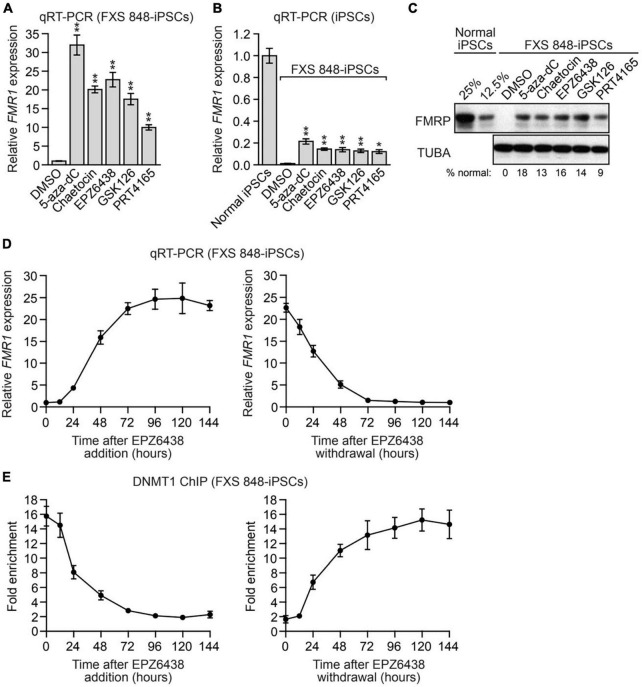
Reactivation of epigenetically silenced *FMR1* by small molecule *FMR1*-SF inhibitors. **(A,B)** qRT-PCR analysis monitoring *FMR1* expression in FXS 848-iPSCs treated with 5-aza-dC, chaetocin, EPZ6438, GSK126, PRT4165 or, as a control, DMSO. The results were normalized to that obtained with DMSO **(A)** or in normal iPSCs **(B)**, which was set to 1. **(C)** Immunoblot analysis monitoring FMRP levels in FXS 848-iPSCs treated with 5-aza-dC, chaetocin, EPZ6438, GSK126, PRT4165 or, as a control, DMSO. The FMRP signal was quantified and normalized to that obtained in normal iPSCs, which was multiplied by the dilution factor and then set to 100%. **(D)** qRT-PCR analysis monitoring *FMR1* expression in FXS 848-iPSCs following EPZ6438 addition (left) or withdrawal (right). **(E)** ChIP analysis monitoring DNMT1 binding to the *FMR1* promoter in FXS 848-iPSCs following EPZ6438 addition (left) or withdrawal (right). Data are represented as mean ± SD (*n* = 3 biological replicates). *P < 0.05, **P < 0.01.

For several of the *FMR1*-SFs we identified there are well-described small molecule inhibitors. Figure 2A shows that epigenetically silenced *FMR1* could be reactivated by treatment with 5-aza- dC, consistent with the results of previous studies (Bar-Nur et al., 2012; Kumari and Usdin, 2014; Tabolacci et al., 2016; Vershkov et al., 2022). Notably, epigenetically silenced *FMR1* was also reactivated following treatment with small molecule inhibitors of EZH2 (EPZ6438, GSK126), SUV39H1 (chaeotocin) and RNF2 (PRT4165). Reactivation was observed by analysis of both *FMR1* mRNA (Figure 2B and Supplementary Figure 6) and FMRP protein (Figure 2C) and was again ∼10–20% of normal levels. Similar results were obtained in FXS SC135-iPSCs (Supplementary Figure 7).

We performed several additional experiments with the EZH2 inhibitor EPZ6438 (also called tazemetostat), which recently received FDA approval for the treatment of certain cancers (Straining and Eighmy, 2022).^1^ The EPZ6438 titration experiment of Supplementary Figure 8 reveals a very good correlation between the loss of EZH2 enzymatic activity, as evidenced by decreased total H3K27me3, and reactivation of epigenetically silenced *FMR1*. The time course experiment of Figure 2D shows that the level of *FMR1* reactivation increased over 96 h. Withdrawal of EPZ6438 resulted in re-silencing of *FMR1*, which again occurred over a time course of ∼96 h. The ChIP experiment of Figure 2E shows that the association of DNMT1 with the epigenetically silenced *FMR1* promoter was well correlated with the kinetics of *FMR1* reactivation following EPZ6438 addition and re-establishment of *FMR1* silencing following EPZ6438 withdrawal. Collectively, these results indicate that both silencing and reactivation of *FMR1* are reversible.

### The *FMR1*-SFs promote epigenetic silencing of *FMR1* in FXS neural progenitor cells and post-mitotic neurons

**FIGURE 3:**
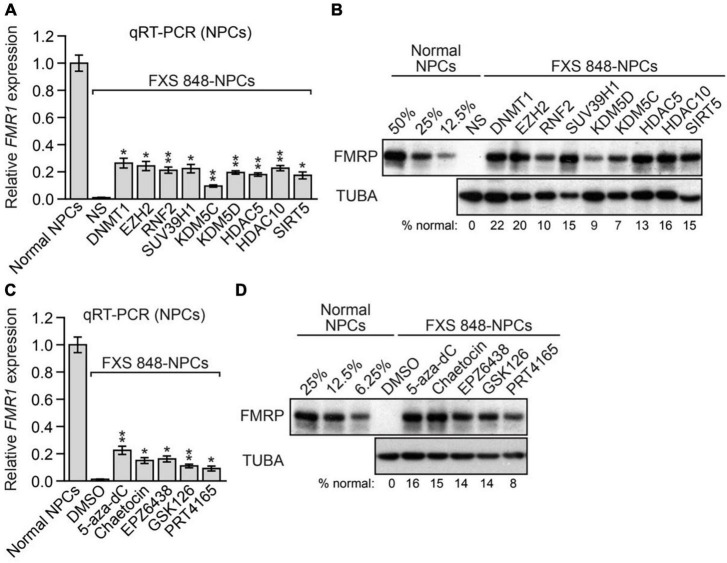
The *FMR1*-SFs promote epigenetic silencing of *FMR1* in FXS NPCs. **(A)** qRT-PCR analysis monitoring *FMR1* expression in FXS 848-NPCs expressing an *FMR1*-SF shRNA. The results were normalized to that obtained in normal NPCs, which was set to 1. **(B)** Immunoblot analysis showing FMRP protein levels in FXS 848-NPCs expressing an *FMR1*-SF shRNA. The FMRP signal was quantified and normalized to that obtained in normal NPCs, which was multiplied by the dilution factor and then set to 100%. **(C)** qRT-PCR analysis monitoring *FMR1* expression in FXS 848-NPCs treated with 5-aza-dC, chaetocin, EPZ6438, GSK126, PRT4165, or, as a control, DMSO. **(D)** Immunoblot analysis monitoring FMRP levels in FXS 848-NPCs treated with 5-aza-dC, chaetocin, EPZ6438, GSK126, PRT4165, or, as a control, DMSO. Data are represented as mean ± SD (*n* = 3 biological replicates). *P < 0.05, **P < 0.01.

The experiments described above were performed in undifferentiated FXS iPSCs. We next asked whether inhibition of the same set of nine *FMR1*-SFs would also reactivate epigenetically silenced *FMR1* in FXS neural progenitor cells (NPCs) and post-mitotic neurons, the latter of which is the cell type most relevant to FXS. For these experiments, we used an FXS NPC cell line that was derived from FXS 848-iPSCs [hereafter called FXS 848-NPCs; (Sheridan et al., 2011)]. Knockdown of any one of the nine *FMR1*-SFs reactivated epigenetically silenced *FMR1* in FXS 848-NPCs at both the mRNA (Figure 3A) and protein (Figure 3B) levels. Epigenetically silenced *FMR1* was also reactivated in FXS 848-NPCs by small molecule inhibitors of *FMR1*-SFs including 5- aza-dC, chaetocin, EPZ6438, GSK126, and PRT4165, at both the mRNA (Figure 3C) and protein (Figure 3D) levels.

**FIGURE 4:**
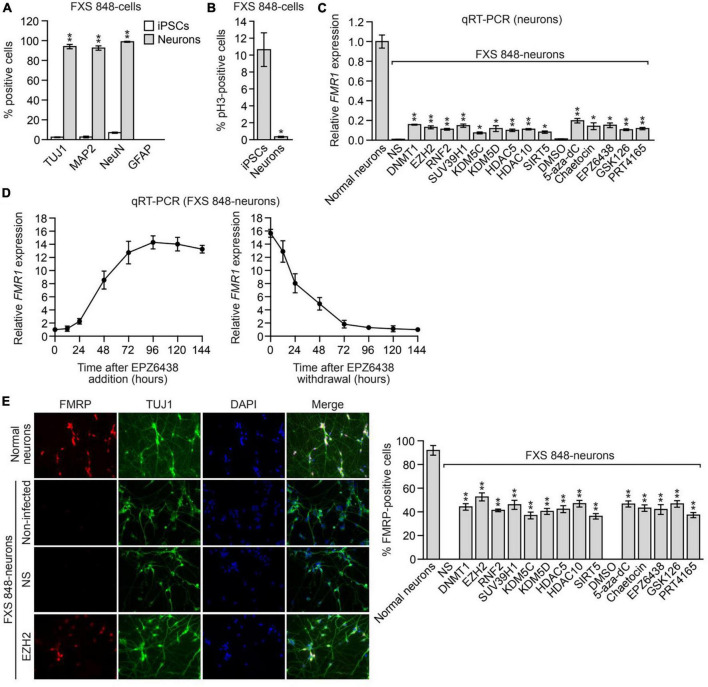
The *FMR1*-SFs promote epigenetic silencing of *FMR1* in FXS post-mitotic neurons. Percentage of TUJ1-, MAP2-, NeuN- and GFAP-positive cells **(A)** or phosphorylated H3- positive cells **(B)** in FXS 848-iPSCs and FXS 848-neurons. Data are represented as mean ± SD (*n* = 3 biological replicates with at least 300 cells analyzed per sample). **(C)** qRT-PCR analysis monitoring *FMR1* expression in FXS 848-neurons expressing an *FMR1*-SF shRNA or treated with a small molecule *FMR1*-SF inhibitor. Data are represented as mean ± SD (*n* = 3 biological replicates). **(D)** qRT-PCR analysis monitoring *FMR1* expression in FXS 848-neurons following EPZ6438 addition (left) or withdrawal (right). Neurons were treated with 5 μM EPZ6438 for 96h, then washed 3 times with PBS, refreshed with neuron growth medium, and collected at different time points for analysis. Data are represented as mean ± SD (*n* = 3 biological replicates). **(E)** Left, ICC monitoring FMRP levels in normal neurons and FXS 848-neurons expressing an NS or EZH2 shRNA, or in non-infected FXS 848-neurons (not expressing an shRNA). Right, Quantification of the percentage of FMRP-positive cells in FXS 848-neurons expressing an *FMR1*-SF shRNA or treated with a small molecule *FMR1*-SF inhibitor. Data are represented as mean ± SD (*n* = 3 biological replicates with at least 300 cells analyzed per sample). *P < 0.05, **P < 0.01.

To derive FXS post-mitotic neurons, FXS 848-iPSCs were transduced with a lentivirus expressing *Neurogenin-1* and *Neurogenin-2* according to published methods (Busskamp et al., 2014); for convenience we refer to these cells as FXS 848-neurons. Neuronal differentiation was assessed and confirmed by staining with neuronal markers including TUJ1, MAP2, and NeuN (Figure 4A and Supplementary Figure 9A). Glial fibrillary acidic protein (GFAP)-positive glial cells were not detected in the FXS 848-neuronal cultures (Figure 4A). As expected, the FXS 848-neurons were post-mitotic as evidenced by the lack of the mitotic marker phosphorylated histone H3 (Figure 4B). Treatment of FXS 848-neurons with an *FMR1*-SF shRNA or small molecule *FMR1*-SF inhibitor reactivated epigenetically silenced *FMR1* (Figure 4C). Similar to what we found in FXS 848-iPSCs (Figure 2D), in FXS 848-neurons both reactivation and silencing of *FMR1* with the EZH2 inhibitor EPZ6438 was reversible (Figure 4D). As expected, EPZ6438 treatment substantially reduced total H3K27me3 levels in FXS 848-neurons (Supplementary Figure 9B).

To rule out the possibility that the *FMR1* reactivation we observed was from contaminating dividing cells, FXS 848-neuronal cultures were treated with a potent inhibitor of DNA synthesis, cytosine arabinoside (Ara-C), which kills proliferating cells (Henriquez et al., 2013). We found that Ara-C treatment had no effect on the ability of *FMR1*-SF shRNAs and small molecule *FMR1*- SF inhibitors to reactivate epigenetically silenced *FMR1* (Supplementary Figure 9C), indicating that the observed *FMR1* reactivation was indeed from post-mitotic cells.

We found it was technically challenging to obtain a sufficient number of iPSC-derived neurons for quantifying FMRP levels by immunoblot analysis. Therefore, as in previous studies (Bar-Nur et al., 2012; Doers et al., 2014), to monitor FMRP levels in neurons, we performed immunocytochemistry (ICC). Figure 4E shows the ICC results following knockdown of a representative *FMR1*-SF, EZH2 (left), the FMRP level from shEZH2 is significantly higher than shNS but much weaker than normal neuron, and a quantitative summary of the results of all *FMR1*- SF shRNAs and small molecule *FMR1*-SF inhibitors (right), which confirm *FMR1* reactivation at the protein level.

We confirmed these results in a second FXS neuronal cell line, FXS SC135-neurons, which were derived by transducing FXS SC135-iPSCs with a lentivirus expressing *Neurogenin 1* and *Neurogenin 2* as described above. Neuronal differentiation was confirmed by staining with neuronal markers including TUJ1 and NeuN (Supplementary Figure 10A), and the FXS SC135-neurons were post- mitotic as evidenced by the lack of phosphorylated histone H3 (Supplementary Figure 10B). Treatment of FXS SC135-neurons with an *FMR1*-SF shRNA or small molecule *FMR1*-SF inhibitor reactivated epigenetically silenced *FMR1* at both the mRNA (Supplementary Figure 10C) and protein (Supplementary Figure 10D) levels, which was unaffected by pre-treatment with Ara-C (Supplementary Figure 10E).

Finally, we sought to confirm our key findings using an isogenic pair of FXS cell lines in which the CGG repeats are either intact (CGG-intact) or have been excised using CRISPR/Cas9- mediated deletion (CGG-excised) (Xie et al., 2016). For these experiments, we monitored *FMR1* reactivation following knockdown or inhibition of a representative *FMR1*-SF, EZH2. Consistent with our results in other FXS cell lines, treatment of FXS (CGG-intact) iPSCs and neurons with EZH2 shRNAs or small molecule EZH2 inhibitors restored *FMR1* mRNA or FMRP protein to ∼10–20% of the levels observed in FXS (CGG-excised) iPSCs and neurons (Supplementary Figures 11A–D).

### *FMR1* reactivation normalizes characteristic molecular abnormalities of FXS neurons

We next performed a series of experiments to determine whether the level of *FMR1* reactivation we obtained was sufficient to normalize characteristic molecular abnormalities of FXS neurons. The transcription factor REST is a master negative regulator of neurogenesis that controls the pool size and timing of differentiation of various neural lineages (Schoenherr and Anderson, 1995; Chen et al., 1998; Covey et al., 2012; Satoh et al., 2013). REST is expressed in embryonic stem cells, NPCs, and non-neuronal cells, where it suppresses neuron-specific genes, and is not expressed in differentiated neurons (Ballas et al., 2005). FMRP helps maintain the levels of a neural-specific miRNA (hsa-mir-382) (Halevy et al., 2015), which is a repressor of REST translation. Thus, in the absence of FMRP, the levels of hsa-mir-382 are decreased, preventing the differentiation- dependent downregulation of REST. The resulting higher levels of REST in FXS neurons lead to the suppression of axonal guidance and other genes important for neural development.

**FIGURE 5:**
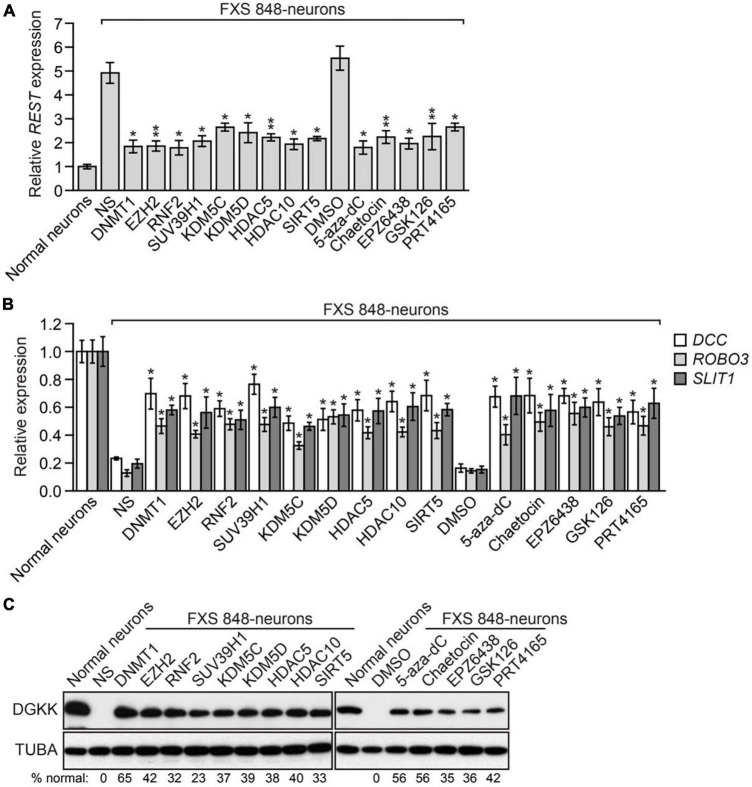
*FMR1* reactivation normalizes molecular abnormalities of FXS neurons. qRT- PCR analysis monitoring expression of *REST* **(A)** or *DCC*, *ROBO3* and *SLIT1* **(B)** in FXS 848- neurons expressing an *FMR1*-SF shRNA or treated with a small molecule *FMR1*-SF inhibitor. The expression of *FMR1* in FXS 848-neurons is shown relative to that in normal neurons, which was set to 1. **(C)** Immunoblot analysis showing DGKK levels in FXS 848-neurons expressing an *FMR1*-SF shRNA or treated with a small molecule *FMR1*-SF inhibitor. DGKK levels in normal neurons are shown. The DGKK signal was quantified and normalized to that obtained in normal neurons, which was set to 100%. Data are represented as mean ± SD (*n* = 3 biological replicates). *P < 0.05, **P < 0.01.

Consistent with previous studies, qRT-PCR analysis revealed that compared to their normal counterparts, FXS 848-neurons contain increased levels of *REST* (Figure 5A) and decreased levels of the REST target axonal guidance genes *DCC*, *ROBO3*, and *SLIT1* (Figure 5B). Treatment of FXS 848-neurons with an *FMR1*-SF shRNA or small molecule *FMR1*-SF inhibitor substantially decreased expression of *REST* (Figure 5A) and increased expression of *DCC*, *ROBO3*, and *SLIT1* (Figure 5B). Similar results were obtained with FXS SC135-neurons (Supplementary Figure 12).

A potential concern of the above results is that the normalized expression of *REST*, *DCC*, *ROBO3*, and *SLIT1* we observed may be an indirect effect resulting from inhibition of an epigenetic regulator rather than a direct effect of *FMR1* reactivation. To address this concern, we first asked whether normalized expression of *REST*, *DCC*, *ROBO3*, and *SLIT1* was dependent on *FMR1*. We found that the decreased expression of *REST* and increased expression of *DCC*, *ROBO3*, and *SLIT1* observed following treatment of FXS 848-neurons with an *FMR1*-SF shRNA or small molecule *FMR1*-SF inhibitor was abrogated by shRNA-mediated knockdown of *FMR1*, indicative of *FMR1* dependence (Supplementary Figures 13A–C). We also asked whether normalization of REST target gene expression following *FMR1* reactivation was due to the decreased levels of REST. We found that the increased expression of *DCC*, *ROBO3*, and *SLIT1* observed following treatment of FXS 848-neurons with an *FMR1*-SF shRNA or small molecule *FMR1*-SF inhibitor was abrogated by ectopic expression of *REST* (Supplementary Figures 13D, E). Collectively, these results demonstrate that the normalized expression of molecular markers following *FMR1*-SF inhibition is due to *FMR1* reactivation and consequent FMRP-mediated down-regulation of *REST*.

FMRP has been shown to physically associate with the mRNA encoding DGKK, a kinase that controls the switch between diacylglycerol and phosphatidic acid signaling pathways (Tabet et al., 2016). The absence of FMRP in *FMR1* knockout mouse cortical neurons results in decreased levels of DGKK, and shRNA-mediated loss of function of *Dgkk* is sufficient to cause dendritic spine abnormalities, synaptic plasticity alterations, and behavioral disorders similar to those observed in *FMR1* knockout mice (Tabet et al., 2016). Notably, ectopic expression of DGKK rescues the dendritic spine defects of *FMR1* knockout neurons (Tabet et al., 2016) and adeno- associated viral vector delivery of DGKK corrects abnormal diacylglycerol and phosphatidic acid homeostasis and behavioral disorders in *FMR1* knockout mice (Habbas et al., 2022). Consistent with these published results, immunoblot analysis showed that DGKK was readily detectable in normal but not in FXS 848-neurons (Figure 5C). Treatment of FXS 848-neurons with an *FMR1*-SF shRNA or small molecule *FMR1*-SF inhibitor substantially increased DGKK levels. The increased levels of DGKK observed following treatment with an *FMR1*-SF shRNA or small molecule *FMR1*-SF inhibitor was abolished upon shRNA-mediated knockdown of *FMR1* confirming the *FMR1*-dependence of this effect (Supplementary Figure 14A). Similar results were obtained with FXS SC135- neurons (Supplementary Figures 14B, C).

### EZH2 inhibition corrects electrophysiological abnormalities in cultured FXS neurons and reactivates *FMR1* expression in human FXS NPCs engrafted within the brains of mice

In the final set of experiments, we elected to focus on the *FMR1*-SF EZH2 because EZH2 inhibition is well tolerated in cultured cells, mice and humans, and, as stated above, because small molecule EZH2 inhibitors are clinically well advanced, with EPZ6438 recently receiving FDA approval for the treatment of certain cancers (see text footnote 1; Straining and Eighmy, 2022).

**FIGURE 6:**
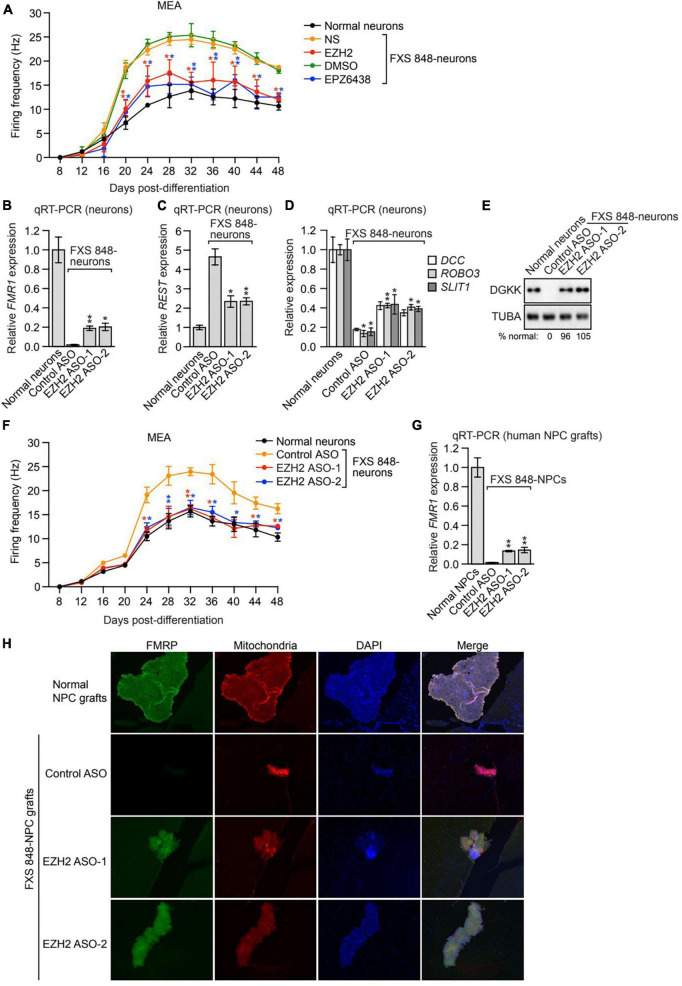
EZH2 inhibition corrects electrophysiological abnormalities in cultured FXS neurons and reactivates *FMR1* expression in human FXS NPCs engrafted within the brains of mice. **(A)** MEA showing firing frequency of FXS 848-neurons expressing an EZH2 shRNA or treated with EPZ6438. The firing frequency of normal neurons is shown as a control. **(B)** qRT-PCR analysis monitoring *FMR1* expression in cultured FXS 848-neurons treated with a control or EZH2 ASO. qRT-PCR analysis monitoring expression of *REST* **(C)** or *DCC*, *ROBO3*, and *SLIT1.* **(D)** in cultured FXS 848-neurons treated with an EZH2 ASO. **(E)** Immunoblot analysis showing DGKK levels in cultured FXS 848-neurons treated with an EZH2 ASO. The DGKK signal was quantified relative to that obtained in normal neurons. **(F)** MEA showing firing frequency of cultured FXS 848-neurons treated with an EZH2 ASO. The firing frequency of normal neurons is shown. **(G)** qRT-PCR analysis monitoring *FMR1* expression in FXS 848-NPC grafts in mice (*n* = 4) treated with an EZH2 or control ASO by ICV injection. **(H)** Representative immunohistochemical images of mouse brain sections (subventricular zone) showing staining for human FMRP (green), human mitochondria (red) and DAPI (blue, total cells from both mouse and human) following treatment with an EZH2 or control ASO. The merged image is shown. Data are generally represented as mean ± SD (*n* = 3 biological replicates), with the exception of **(G)**, for which *n* = 4 biological replicates). *P < 0.05, **P < 0.01.

Previous studies have shown that loss of FMRP results in the characteristic electrophysiological abnormality of neuronal hyperexcitability [reviewed in (Contractor et al., 2015)]. Reactivation of *FMR1* by targeted demethylation of the CGG repeats has been shown to ameliorate the hyperexcitability of FXS neurons (Liu et al., 2018). We therefore asked whether *FMR1* reactivation resulting from inhibition of the *FMR1*-SF EZH2 could normalize the hyperexcitability of FXS neurons. In these experiments, we measured neuronal hyperexcitability using multielectrode arrays (MEAs). In brief, FXS 848-neurons were treated with either an EZH2 shRNA or the small molecule EZH2 inhibitor EPZ6438 and cultured on MEAs, and the spontaneous firing frequency, a measure of excitability, was monitored over a 48-day time course. Consistent with previous studies (Liu et al., 2018), the MEA results revealed that compared to their normal counterparts, FXS 848-neurons displayed increased firing frequency, indicative of hyperexcitability (Figure 6A). Treatment of FXS 848-neurons with an EZH2 shRNA or EPZ6438 substantially decreased the firing frequency. Thus, the level of *FMR1* reactivation obtained by knockdown or pharmacological inhibition of EZH2 is sufficient to correct the hyperexcitability of FXS neurons.

Currently, there is no FXS mouse model to directly analyze the therapeutic benefit of *FMR1* reactivation, because incorporation of the CGG repeat expansion in mice fails to recapitulate the DNA hypermethylation and transcriptional silencing of *FMR1* that occurs in human cells (Dahlhaus, 2018). As an alternative approach, two groups have reported the development of a mouse engraftment model to analyze *FMR1* reactivation in the central nervous system (CNS) (Liu et al., 2018; Vershkov et al., 2019). In this model, FXS NPCs are stereotaxically injected into the brains of mice, and the grafts are subsequently analyzed for human *FMR1/*FMRP expression.

We therefore sought to test whether EZH2 inhibition could reactivate epigenetically silenced *FMR1* in this mouse engraftment model. Current EZH2 inhibitors do not efficiently cross the blood-brain barrier (Zhang et al., 2015), which limits their potential use as FXS therapeutics. Recently, antisense oligonucleotide (ASO)-based approaches have been developed as effective treatment options for certain CNS disorders (Bennett et al., 2019). Accordingly, we derived two individual EZH2 ASOs that efficaciously reduced *EZH2* expression (Supplementary Figure 15A) and decreased H3K27me3 levels in FXS 848-neurons (Supplementary Figure 15B). Treatment of cultured FXS 848-neurons with either of the EZH2 ASOs reactivated *FMR1* to ∼20% of normal levels, comparable to EZH2 small molecule inhibitors (Figure 6B). As expected, the EZH2 ASOs also substantially decreased expression of *REST* (Figure 6C), increased expression of *DCC*, *ROBO3* and *SLIT1* (Figure 6D), and increased DGKK levels (Figure 6E). Furthermore, the EZH2 ASOs corrected the hyperexcitability of FXS 848-neurons (Figure 6F). Similar results were obtained in neurons derived from the isogenic FXS (CGG-excised/intact) iPSC lines (Supplementary Figures 15C–G), although the increased expression of *DCC*, *ROBO3*, and *SLIT1* is not significant may due to cell line difference.

To test whether EZH2 ASOs could reactivate *FMR1 in vivo*, FXS 848-NPCs were stereotaxically injected into the cerebral lateral ventricles of mice. Three days after transplantation, mice received an intracerebroventricular injection of an *EZH2* ASO, and 7 days later, mice were sacrificed and the brain grafts collected and dissected. Analysis by qRT-PCR using primers specific for the human *FMR1* transcript (Vershkov et al., 2019) showed that EZH2 ASOs reactivated expression of *FMR1* to ∼15% of normal levels (Figure 6G). Furthermore, immunofluorescence staining of mouse brain sections with a human-specific anti-FMRP antibody revealed that EZH2 ASO treatment also resulted in increased FMRP levels (Figure 6H). Collectively, these results demonstrate that EZH2 ASOs can reactivate *FMR1 in vivo*.

## Discussion

Here, using a candidate-based shRNA screen, we identified nine factors (*FMR1*-SFs) that are required for epigenetic silencing of *FMR1*. One of the *FMR1*-SFs was the H3K27 methyltransferase EZH2, which became the major focus of our study. We found that EZH2 was recruited to epigenetically silenced *FMR1* and that inhibition of EZH2 using shRNAs, small molecule inhibitors or ASOs reactivated *FMR1* in multiple, independent FXS iPSC lines, NPCs and post-mitotic neurons. The level of *FMR1* reactivation following EZH2 inhibition was not complete but rather 10–20% that of normal levels. Notably, however, this level of *FMR1* reactivation was sufficient to substantially correct characteristic electrophysiological and molecular abnormalities of FXS neurons including altered levels of REST, axonal guidance proteins and DGKK. Of particular significance is correction of the DGKK deficiency, which has been shown to be responsible for disease features of FXS neurons such as dendritic spine defects and behavioral disorders (Tabet et al., 2016). Our finding that partial *FMR1* reactivation substantially normalized FXS neurons is consistent with several previous observations suggesting that even intermediate levels of FMRP are sufficient to confer a normal phenotype in humans and mice. For example, asymptomatic carriers of a premutation (55-200 CGG repeats) (Sheridan et al., 2011) and rare asymptomatic individuals who have an *FMR1* full mutation but *FMR1* is not silenced (Tabolacci et al., 2008b) have FMRP levels that are only ∼20% that of normal individuals. Moreover, ectopic expression of FMRP to only ∼35% of normal levels in central nervous system neurons of *FMR1* knockout mice results in significant phenotypic rescue (Arsenault et al., 2016).

The epigenetic silenced *FMR1* gene exhibits the characteristic features of heterochromatin, such as DNA hypermethylation, the acquisition of repressive histone modifications like H3 lysine 9 trimethylation (H3K9me3), H3 lysine 27 trimethylation (H3K27me3), and H4 lysine 20 trimethylation (H4K20me3), and the loss of activating histone modifications like H3 lysine 4 trimethylation (H3K4me3) and H2A/H2B/H3/H4 acetylation (Oberle et al., 1991; Coffee et al., 1999, 2002; Pietrobono et al., 2005; Tabolacci et al., 2005, 2008a; Kumari and Usdin, 2010). Surprisingly little is known about the precise mechanisms that write, read, or delete the epigenetic imprints on the silenced *FMR1* promoter. In this study, through candidate- based RNAi screen, we have identified EZH2 for K3K27 methylation, RNF2 for ubiquitination of histone H2A, SUV39H1 for histone K3K9 methylation, KDM5C, KDM5D for H3K4 demethylation, HDAC5 and HDAC10 for histone deacetylation, and DNMT1 for DNA methylation. These findings are summarized in Supplementary Figure 1. Except for SIRT5, other 8 FMR1-SF are bound to FMR1 locus. We believe these 8 factors are directly responsible for increased repressive marks at *FMR1* locus, and the 8 factors act in a concerted way, the other 7 factors works upstream of DNMT1, as knockdown of other 7 factors eliminates DNA methylation at *FMR1* locus (Figure 1D). An instructive model posits that epigenetic silencing of tumor suppressor in cancers occurs through a specific pathway, comprising a defined set of components, initiated by an oncoprotein (Fang et al., 2014, 2016; Serra et al., 2014; Struhl, 2014). In FXS, it is likely that a transcription factor binds to FMR1 locus in a sequence specific way, which recruits the 8 FMR-SFs to initiate the modification of increased repressive marks and final epigenetic silencing of *FMR1*.

In this study, the shRNA or small molecules of FMR1-SF reactivates silences FMR1, but the FMRP levels are much weaker than normal neurons, and the percentages of FMRP positive FXS neurons are lower than normal neurons from ICC (Figure 4E). Furthermore, it is evident that FMRP reactivation and restoration are heterogeneous, as both FMRP positive and negative FXS neurons can be observed in the same picture and treatment. Despite the fact that a large portion of neurons—more than 90%—are FMRP positive, this heterogeneity is also seen in normal neurons. Other groups have also previously discovered this heterogeneity in human neurons (Bar-Nur et al., 2012; Doers et al., 2014). The distinct pattern of FMRP immunostaining from homogenous neuron cell type was also reported to other species *in vivo*, the variation of FMRP intensity across neurons were quantified by the z-score of FMRP immunostaining from −2 to 2, and the neurons with a z- score above 2 exhibited FMRP aggregation over time (Wang et al., 2014; Yu et al., 2021). First this heterogeneity could be biased from image plane, full 3D scanning of whole cell will reduce the bias. According to a recent study, sensory input regulates the FMRP protein’s dynamics in neurons as well as its level, cellular localization, granular structure, and immunostaining intensity over time (Yu et al., 2021). The observed heterogeneity of FMRP restoration may be explained by the dynamic nature of normal FMRP functions in relation to cellular and synaptic activity.

We note that in contrast to our findings, a previous study reported that inhibition of EZH2 did not reactivate epigenetically silenced *FMR1* in FXS cells, although EZH2 inhibition did delay re-silencing of *FMR1* following 5-aza-dC treatment and withdrawal (Kumari and Usdin, 2016). One possible reason for the failure of this previous study to observe *FMR1* reactivation by EZH2 inhibitors is that we have found the level of *FMR1* reactivation induced by EZH2 inhibitors is highly cell density-dependent and is substantially reduced at high cell density (Supplementary Figure 16). Notably, previous studies have shown that the transcriptional activity of many genes is cell density- dependent (Kim et al., 2015). Perhaps the experiments in the previous study were carried out at a cell density that was too high to obtain *FMR1* reactivation upon EZH2 inhibition. An alternative explanation is differences among the cell lines, such as the lengths of CGG repeats and extent of DNA methylation, as previously noted (Kumari et al., 2019).

Previous studies have shown that DNA demethylation can reactivate epigenetically silenced *FMR1*. For example, targeted demethylation of the *FMR1* CGG repeats by a dCas9-Tet1 fusion protein was found to reactivate *FMR1* (Liu et al., 2018). However, from a clinical perspective it is currently not feasible, by gene therapy or other approaches, to deliver the large dCas9-Tet1 fusion protein to a sufficient number of CNS neurons to have a therapeutic benefit. In addition, several previous studies have shown reactivation of *FMR1* by DNMT inhibitors such as 5-aza- dC (Bar-Nur et al., 2012; Kumari and Usdin, 2014; Tabolacci et al., 2016) in cultured cells and following systemic treatment of mice in the human NPC mouse brain engraftment model (Vershkov et al., 2019). However, 5-aza-dC is poorly CNS penetrant and has significant toxicity. For example, following systemic administration, the concentration of 5-aza-dC in the peripheral circulation is approximately 100-fold higher than that in the CNS, which would result in unacceptable toxicity (Lester McCully et al., 2020).

*FMR1*’s expansion of more than 200 CGG repeats causes transcriptional silence and FMRP loss. The length of the CGG repeat is inversely connected with *FMR1* repression. The *FMR1* gene, which has been epigenetically silenced, exhibits the usual characteristics of heterochromatin, such as increased repressive histone modifications and DNA hypermethylation. The recent work from Liu et al. (2018) *FMR1* was once again persistently expressed in FXS iPSCs after targeted demethylation of the CGG expansion by dCas9-Tet1, changed the upstream *FMR1* promoter’s heterochromatin status to an active chromatin state. Neurons produced from methylation altered FXS iPSCs corrected the electrophysiological defects and restored a wild-type behavior onto the mutant neurons (Liu et al., 2018). The FXS-FS shRNA, small compounds and ASO in this work also reduce the DNA methylation and repressive histone marks, restores active chromatin state at *FMR1* promoter and *FMR1* expression in FXS iPSC and neurons. Our findings expand the possible intervention of FXS to small compounds and ASO, and they are in line with the study by Liu et al. (2018).

A potential concern of *FMR1* transcriptional upregulation as a therapeutic approach is that the factors responsible for *FMR1* repression act upon multiple genes and thus their inhibition will affect the expression of genes other than *FMR1*. However, the fact that expression of other genes will be altered does not mean that inhibition of an FXN-RF is unsafe. EZH2 is a core component of one of the most studied chromatin regulatory factors catalyzed the mono-, di-, and tri-methylation of histone H3K27me3 (Margueron and Reinberg, 2011). Analysis of the genome reveals that EZH2 inhibition causes a global loss of H3K27me3. At the same time, a significant fraction of H3K27me3 is retained at a small subset of genomic loci, along with the accumulation of PRC2, at genomic loci that had a high baseline level of H3K27me3 in the studies that included EZH2 inhibition (Xu et al., 2015), and deletion of EZH2 in a mouse model (Popovic et al., 2014). Recent clinical trials have demonstrated that the EZH2 inhibitor Tazemetostat is a safe and effective oral treatment for follicular lymphoma and epithelioid sarcoma, with manageable side effects (Gounder et al., 2020; Morschhauser et al., 2020). The surprisingly wide variance in gene expression profiles across healthy individuals may be one reason for the apparent safety of blocking particular epigenetic regulators, like EZH2 (Cheung et al., 2003; Storey et al., 2007; Reinhold et al., 2012; Lappalainen et al., 2013). For instance, it has been calculated that in normal individuals, up to ∼83% of genes have variable expression (Storey et al., 2007. Therefore, it would seem that humans have a strong buffering mechanism against variations in gene expression and are therefore able to tolerate modifications in gene expression brought on by pharmacological agents.

Here we have shown that EZH2 ASOs reactivate *FMR1* expression and substantially correct characteristic molecular and electrophysiological abnormalities in cultured FXS neurons, and reactivate *FMR1* in human FXS NPCs engrafted within the brains of mice. ASO therapeutics have received considerable attention for treatment of certain CNS disorders (Wurster and Ludolph, 2018), based largely on the success of the intrathecal administration of the ASO nusinersen to treat spinal muscular atrophy (Goodkey et al., 2018). ASOs have a number of attractive features as CNS therapeutics, including rapid distribution throughout the spinal cord and into most regions of the brain following intrathecal injection, and relatively long half-life in the CNS tissues allowing for infrequent administration (Bennett et al., 2019). Collectively, our results establish EZH2 inhibition in general, and EZH2 ASOs in particular, as a potential and feasible therapeutic approach for FXS.

In FXS patients, the transcriptional silencing of the *FMR1* gene is initiated by an expansion of a naturally occurring CGG repeat in the 5′ UTR of the *FMR1* gene, to more than 200 units. The hypermethylation of FMR1 locus correlates the CGG repeat length. One limitation of this study lies in that the CGG repeat numbers were not constantly tracked in every step. Despite the fact that 400–900 repetitions have been found (Sheridan et al., 2011). Furthermore, as indicated by the bisulfite sequencing in Figure 1D, the DMSO or shNS FXS 848 iPSC exhibits hypermethylation at the *FMR1* promoter. The hairpins of the selected candidates decrease DNA methylation, which is correlated with elevated amounts of *FMR1* mRNA and protein. A restricted number of clones for DNA methylation analysis, a restricted section with engrafted NPC from acquired mouse brain tissues and no intensity measurement of FMRP immunostaining in ICC are further limitations of this study. These restrictions make the conclusion less perfect.

## Materials and methods study design

The objectives of this study were to identify epigenetic factors that promote *FMR1* silencing (*FMR1*-SFs) and efficacious small molecule *FMR1*-SF inhibitors, determine whether the level of *FMR1* reactivation obtained with biological or pharmacological *FMR1*-SF inhibitors can normalize the dysfunctional phenotypes of human FXS neurons, and establish whether *FMR1*-SF inhibition is a clinically viable therapeutic strategy for reactivating *FMR1 in vivo*. The study used previously described iPSC and NPC lines derived from human FXS patients, iPSC-derived neurons, and mouse models. The study consisted of a series of controlled laboratory experiments and measured multiple parameters including gene expression, protein levels, promoter methylation and occupancy, and neuron firing frequency as described below. For the animal experiments, mice were randomly allocated to each experimental group, and were subsequently analyzed in a non- blinded fashion. No data outliers were excluded. Animal sample sizes were selected based on precedent established from previous publications. All other quantitative data were collected from experiments performed in at least triplicate.

### Cell culture

BJ1-iPS4 cells (normal iPSCs), FXS 848-iPS3 cells (FXS 848-iPSCs), and NPCs derived from normal 8330-iPS8 cells (normal NPCs) or FXS 848-iPS3 cells (FXS 848-NPCs) passage 4–6 (Sheridan et al., 2011) were kindly provided by Stephen J. Haggarty (Harvard Medical School). For this study, the FXS 848-iPS3 passage 6–8 were used for shRNA, small molecule treatment and neuron differentiation. In The FXS iPSC line SC135 (FXS SC135-iPSCs) (Brick et al., 2014) was kindly provided by Marius Wernig (Stanford School of Medicine). The isogenic FXS (CGG- excised/intact) iPSC lines (Xie et al., 2016) were kindly provided by Peng Jin (Emory University School of Medicine). Normal and FXS iPSCs and NPCs were authenticated by qRT-PCR analysis to validate the expected *FMR1* expression status, and by PCR to confirm the expected CGG repeat length in the *FMR1* 5′ UTR. Upon receipt, cells were tested for mycoplasma contamination and found to be negative. iPSCs were cultured in mTeSR1 medium (STEMCELL Technologies) on matrigel-coated plates. NPCs were maintained in neural expansion medium as previously described (Sheridan et al., 2011). Neurons were induced from normal iPSCs, FXS 848-iPSCs or FXS SC135 iPSCs by expression of reverse tetracycline transactivator (rTA3), *Neurogenin-1* and *Neurogenin-2*, and cultured in mTeSR1 and neuron growth medium as previously described (Busskamp et al., 2014; Lam et al., 2017).

### RNAi screen

The 162 shRNAs (listed in Supplementary Table 1) from The RNAi Consortium (TRC) and shRNA^mir^ (pGIPZ) lentiviral human shRNA libraries (Thermo Fisher Scientific) were obtained through the University of Massachusetts RNAi Core Facility and packaged into lentiviruses. FXS 848-iPSCs were seeded in 12-well plates at a density of 1x10^4^ cells/well and transduced with 200 μl lentivirus (at MOI 5) with 10 μg/ml Polybrene (Qiagen) overnight. Two days later, cells were selected with 1.5 μg/ml puromycin for 3 days. Cells were split at days 12 and 18, and harvested at day 20 for analysis of *FMR1* and target gene expression. Based on this analysis, the two most efficacious shRNAs that increased *FMR1* expression and decreased mRNA levels of the target gene were selected for each *FMR1*-SF (indicated in Supplementary Table 1).

### shRNA treatment

iPSCs were seeded at 1x10^4^ cells/well in 12-well plates at 5% confluency, NPCs were seeded at 5x10^4^ cells/well in 12-well plates at 10% confluency, and neurons (at day 4 of the differentiation process) were seeded at 1x10^5^ cells/well in 6-well plates at 40% confluency, and transduced overnight with 200–1000 μl lentivirus (at MOI 5) expressing an *FMR1*-SF shRNA. Two days later, cells were selected with 1.5 μg/ml puromycin for 3 days. iPSCs and NPCs were split at days 12 and 18 at 60–80% confluency, re-seeded at 10% confluency for iPSC and 20% for NPC, and harvested at day 20 at 20% confluency for iPSCs and 30% confluency for NPCs for subsequent experiments (i.e., qRT-PCR, immunoblotting, bisulfite sequencing, and immunofluorescence). Neurons were harvested at day 12 at 40–50% confluency for subsequent experiments.

### Chemical treatment

iPSCs, NPCs and neurons were seeded as described above and 24 h later treated with the following small molecule inhibitors: 5-aza-2′-deoxycytidine (Sigma) at 1 μM and refresh every 24 h to 96 h, chaetocin (Cayman Chemical) at 0.5 μM, EPZ6438 (Cayman Chemical) at 5 μM, GSK126 (APExBIO) at 5 μM, and PRT4165 (Tocris Bioscience) at 5 μM for 96 h. 5-aza-2′-deoxycytidine and chaetocin are toxic to cells, there are 10–30% cell death. iPSCs, NPCs and neurons were harvested at the confluency described above for all subsequent experiments. For the EPZ6438 addition experiments of Figures 2D, 4D, FXS iPSCs or neurons were collected at different time points following incubation with 5 μM EPZ6438. For the EPZ6438 withdrawal experiments, cells were treated with 5 μM EPZ6438 for 96 h, then washed 3 times with PBS, refreshed with mTESR1 medium (for iPSCs) or neuron growth medium (for neurons), and collected at different time points for analysis. For the EPZ6438 titration experiments of Supplementary Figures 2, 8, 9, FXS iPSCs or neurons were treated with varying doses of EPZ6438 (0.001, 0.01, 0.1, 1, or 10 μM) for 96 h. For the cell density dependency experiment of Supplementary Figure 16, FXS iPSCs were seeded at varying densities, and 24 h later treated with 1 μM EPZ6438 for 72 h. For Ara-C treatment, neurons were incubated with 2 μM cytosine arabinoside (Sigma), or water as a control, for 48 h prior to shRNA knockdown or small molecule treatment.

### qRT-PCR

Total RNA was isolated using TRIZOL (Invitrogen) at day 20 or other time points following shRNA lentivirus transduction, puromycin selection or small molecules treatment, or ASO transfection. Reverse transcription was performed using SuperScript II Reverse Transcriptase (Invitrogen) as per the manufacturer’s instructions, followed by quantitative real-time PCR using Platinum SYBR Green qPCR SuperMix-UDG with Rox (Invitrogen). Gene-specific primers are listed in Supplementary Table 2. The Taqman assay was performed using Taqman Fast Advanced Master Mix (Thermo Fisher Scientific) and probes for *FMR1* (Hs00924547_m1) and *ACTB* (Hs99999903_m1), all from Thermo Fisher, according to the manufacturer’s instructions. The Taqman assay for *FMR1* was only used in Supplementary Figure 3, for other figures mRNA levels were determined with SYBR Green and primer in Supplementary Table 2 and with hRPL41 as endogenous control. The experiments were performed in biological triplicates, each with three technical triplicates.

### Immunoblotting

Protein extracts were prepared by lysis in a buffer containing 50 mM Tris–HCl (pH 7.4), 0.1% Triton X-100, 5 mM EDTA, 250 mM NaCl, 50 mM NaF, 0.1 mM Na3VO4, and protease inhibitors (Roche). Blots were probed with an antibody recognizing FMRP (Abcepta, AP6879A), DGKK (Abcam, ab111042), α-tubulin (Sigma, F2168), EZH2 (Fortislife, A304-196A), H3K27me3 (Cell Signaling, 9733), KDM5C (Fortislife, A301-034A), or KDM5D (Fortislife, A301-751A). The FMRP and DGKK signals were quantified using Image J software (NIH), and normalized to α- tubulin levels as previously described (Sheng et al., 2011).

### Bisulfite sequencing

Bisulfite modification was carried out using an EpiTect Bisulfite Kit (Qiagen) followed by PCR amplification as previously described (de Esch et al., 2014). Twenty independent clones were initially sequenced from the PCR product within each cell line, of which six representative clones are displayed in Figure 1D. For quantification, clones with strong sequencing signals (12–16 clones) were analyzed and the percent methylation at each CpG was calculated.

### ChIP assay

ChIP assays were performed as previously described (Gazin et al., 2007) using the following antibodies: DNMT1 (Novus Biologics, AF6110), EZH2 (Fortislife, A304-196A), SUV39H1 (Fortislife, A302-127A), RNF2 (Fortislife, A302-869A), KDM5C (Fortislife, A301-034A) or KDM5D (Fortislife, A301-751A), HDAC5 (Fortislife, 303-464A), and HDAC10 (Sigma, H3413). Briefly twenty millions iPSC cells were harvested and crosslinked with 1% formaldehyde. Extraction and sonication of nuclei were conducted as in Gazin et al. (2007). In the next step, 10 μL of antibody was added to each sample to immunoprecipitate protein DNA complexes. Following reverse-crosslinking and DNA purification, qPCR was performed to quantitate the ChIP products (see Supplementary Table 2 for primers). Samples were quantified as percentage of input, and then normalized to an irrelevant region in the genome (3.2 kb upstream from the transcription start site of *GCLC*) as described (Serra et al., 2014). Fold enrichment was calculated by setting the IgG control IP sample to a value of 1.

### Immunofluorescence

iPSCs, and neurons were fixed with 4% paraformaldehyde in PBS for 10 min, blocked with 10% normal goat serum (Vector Laboratories), and then stained with TUJ1 (Covance), FMRP (Abgent), MAP2 (Cell Signaling), NeuN (Biolegend), GFAP (Abgent), or phosphorylated histone H3 (Abcam) antibodies for 1 h at room temperature. Cells were then rinsed several times with PBS, incubated with Alexa 488- or Alexa 594-conjugated donkey anti-mouse or anti-rabbit secondary antibody (Molecular Probes) and DAPI (Molecular Probes) in the appropriate buffer for 1 h at room temperature. After several more rinses, cells were mounted with Vectashield (Vector Laboratories) and imaged with a Zeiss Imager Z2 microscope equipped with a Zeiss Axiocam digital camera. Images were acquired, and background signal was subtracted, using AxioVision Rel 4.8 software. FMRP or phosphorylated H3 positively stained cells and DAPI- stained nuclei were counted in an automated, blinded fashion using ImageJ. Briefly, the images were converted to grayscale, the lower threshold was set at 20 for FMRP, 82 for phosphorylated H3, and 24 for DAPI, and the Analyze Particle feature was used to obtain the total cell counts. The percentage of cells with positive staining were determined by counting at least 300 cells/nuclei per sample in three separate experiments (for a total of 900 cells/nuclei).

### Restoration of molecular abnormalities by *FMR1* knockdown or ectopic REST expression

Neurons (at day 4 of the differentiation process) were seeded at 1x10^5^ cells/well in 6-well plates at 40% confluency and transduced overnight with lentivirus (200 μl at MOI > 5) expressing an NS or *FMR1*-SF shRNA. Cells were selected with 0.5 μg/ml puromycin for 3 days, and then transduced overnight with a lentivirus (400 μl at MOI 5) expressing an NS or *FMR1* shRNA (TRCN0000059762), or expressing empty vector (pLIX_403, Addgene Plasmid #41395) or REST (pLIX-REST, Addgene Plasmid #91896). Cells were harvested for analysis by qRT-PCR or immunoblot at day 16 post-differentiation at 50% confluency. For small molecule *FMR1*-SF inhibitor treatment, neurons (at day 4 of the differentiation process) were first transduced overnight with a lentivirus (200 μl at MOI > 5) expressing an NS or *FMR1* shRNA, or expressing empty vector or REST, selected with 0.5 μg/ml puromycin for 2 days, and then incubated with a small molecule inhibitor (1 μM 5-aza-2′-deoxycytidine, 0.5 μM chaetocin, 5 μM EPZ6438, 5 μM GSK126 or 5 μM PRT4165) for 96 h. Cells were harvested for analysis by qRT-PCR or immunoblot at day 11 post-differentiation at 50% confluency.

### MEA assay

For shRNA treatment, neurons (at day 4 of the differentiation process) were seeded at 1x10^5^ cells/well in 6-well plates at 40% confluency, transduced overnight with 200 μl lentivirus (at MOI 5) expressing an NS or EZH2 shRNA, and 2 days later selected with 0.5 μg/ml puromycin for 3 days. Following shRNA treatment, 8x10^4^ neurons were overlaid at 90–100% confluency in 24- well MEA plates that had been coated with polyethyleneimine and laminin and seeded 3 days prior with 4x10^4^ rat astrocytes (Thermo Fisher). Thereafter, 80% of the medium was refreshed every 8 days. For small molecule inhibitor treatment, neurons were overlaid on MEA plates and incubated with DMSO or 5 μM EPZ6438 for 4 days. Thereafter, 80% of the medium was refreshed at day 5 without inhibitor, at day 10 with a lower inhibitor concentration (1 μM EPZ6438), and then subsequently every 8 days alternatingly with or without inhibitor. For ASO treatment, neurons were incubated with 1 μM control or EZH2 ASO (see below), and 80% of the medium (with ASO) was refreshed every 8 days.

Starting on the second day of co-culture (at day 8 of the differentiation process), MEA assays were performed every 4 days using a MED64 Presto MEA system (Alpha MED Scientific Inc.) according to the manufacturer’s instructions. Recordings of spontaneous activities were performed using a 3-kHz two-pole Butterworth low pass filter. Experiments were performed in biological triplicate.

### Antisense oligonucleotide design, synthesis and treatment

Conserved regions between the human and mouse EZH2 genes were identified by bioinformatic analysis, and a series of 10 locked nucleic acid (LNA) ASOs targeting these conserved regions were designed using the LNCASO web server.^2^ The LNA ASOs were tested for efficacy in 293T cells by monitoring expression of *EZH2* by qRT-PCR. Derivatives of the most efficacious EZH2 ASO were re-synthesized with 2′-O-methoxyethyl-RNA (MOE) modification and have the following sequences: EZH2 ASO1, &G*&T&C&T& A*C*A*T*G*T*T*T*T*&G&G&T&C*&C, and EZH2 ASO2, &T* &G&T&C&T*A*C*A*T*G*T*T*T*T*&G&G&T&C*&C (where “&” represents MOE modification, and “*” represents phosphorothioate linkage). A non-targeting control ASO was also synthesized with the sequence &C*&C*&T*&A*&T*A*G*G* A*C*T*A*T*C*C*&A*&G*&G*&A*&A. ASOs were synthesized as MOE gapmers (i.e., 5 MOE nucleotides, 8–10 DNA nucleotides, 5 MOE nucleotides) using standard phosphoramidite methods on a Dr. Oligo 48 synthesizer (Biolytic). Phosphoramidites and other standard reagents were purchased from ChemGenes. Coupling times for MOE nucleotides were extended to 2 min. Oligonucleotides were cleaved and deprotected in concentrated aqueous ammonia at 55°C for 16 h. ASOs were characterized by LC-MS analysis using an Agilent Q-TOF LC-MS instrument and were desalted using Amicon ultrafiltration columns (3-kDa cutoff). For ASO treatment, neurons were incubated with 1 μM control or EZH2 ASO for 3 weeks, and 80% of the medium (with ASO) was refreshed every 7 days.

### Animal experiments

All mouse studies were performed in accordance with the Guide for the Care and Use of Laboratory Animals from NIH, and protocols (A2060) approved by the UMMS Institutional Animal Care and Use Committee (IACUC). Mice were randomly allocated to each group. No blinding was done as animal groups were identified by tagging and labeling the cages with the cells/ASOs injected.

C57BL/6J mice (aged 6–9 months, *n* = 4 per group) were anesthetized and placed in a rodent stereotaxic frame (Stoelting), and intracerebroventricular (ICV) injections were performed as previously described (Vershkov et al., 2019). Briefly, the skull was exposed by a small longitudinal incision (<1 cm) along the midline, the periosteum was removed from the surgical area, and small burr holes (<1 mm) were made in each hemisphere using a high-speed drill at the stereotaxic coordinates for ICV injection (X: ±1 mm; Y: −.4 mm; Z: −1.6 mm). NPCs (5x10^4^ in 5 μl volume) were injected slowly into each site using an UltramicroPump (World Precision Instruments) to drive a Hamilton Syringe attached to a 31-gauge steel needle (Hamilton). Staples were used to close the incision and mice were allowed to recover. Three days after NPC transplantation, the initial incision was reopened and 30 pM control or EZH2 ASO was injected into the same site. Seven days after the treatment, mice were sacrificed and the subventricular region was manually dissected for RNA and immunohistochemistry analysis. For RNA analysis, the tissue was mechanically disrupted, and RNA was extracted using a NucleoSpin RNA Plus Kit (Macherey Nagel) and analyzed for *FMR1* expression using primers specific for the human *FMR1* gene (Vershkov et al., 2019). For immunohistochemical staining, brain tissues were fixed in paraformaldehyde, embedded in paraffin and sectioned at a thickness of 10 μm. The immunolabeling was performed by the Morphology Core at the University of Massachusetts Chan Medical School. Briefly, following deparaffinization (60°C for 30 min, xylene for 10 min, 100% ethanol 20 dips, 95% ethanol 10 dips, 75% ethanol 10 dips, H_2_O 1 min), brain sections were processed for antigen retrieval with citrate acid (pH 6.0), blocked with 5% milk in TBS (wt/vol, pH 7.4) at 23–25°C and incubated at 4°C overnight in TBS containing 5% milk with a mouse antibody to human FMRP (AbFrontier, YF-MA10356, 1:500) or a rabbit monoclonal antibody to human mitochondria (Millipore, 1:200). The next day, sections were washed in PBS (pH 7.4) three times, 5 min each, and incubated in secondary antibodies (1: 400) for 1 h at 23–25°C. Sections were also stained with DAPI (1:10,000; Molecular Probes). All images were obtained using a Zeiss AXIO Imager Z2 microscope.

### Statistical analysis

All quantitative data were collected from experiments performed at least three independent times. The results of three technical or biological replicates are shown and expressed as mean ± SD. Differences between groups were assayed using One-way ANOVA with a Dunnett test using Microsoft Excel. Significant differences were considered when *P* < 0.05. Data normalization, if applicable, is described in the relevant figure legend.

## Data availability statement

The raw data supporting the conclusions of this article will be made available by the authors, without undue reservation.

## Ethics statement

The animal study was approved by the UMMS Institutional Animal Care and Use Committee (IACUC). The study was conducted in accordance with the local legislation and institutional requirements.

## Author contributions

MF: Conceptualization, Data curation, Formal Analysis, Funding acquisition, Investigation, Methodology, Supervision, Writing – original draft, Writing – review and editing. SD: Data curation, Funding acquisition, Visualization, Writing – review and editing. PK: Data curation, Investigation, Writing – review and editing. FW: Investigation, Methodology, Resources, Writing – review and editing. PR: Investigation, Methodology, Resources, Writing – review and editing. SB: Investigation, Methodology, Writing – review and editing. C-MV: Methodology, Resources, Writing – review and editing. MS-E: Investigation, Methodology, Resources, Writing – review and editing. JW: Methodology, Resources, Writing – review and editing. MG: Conceptualization, Funding acquisition, Resources, Supervision, Writing – review and editing.

## Footnotes

1
https://www.fda.gov/drugs/fda-granted-accelerated-approval-tazemetostat-follicular-lymphoma

2
https://iomics.ugent.be/lncaso
