# Supplementary figures and images for: EZH2 inhibition reactivates epigenetically silenced FMR1 and normalizes molecular and electrophysiological abnormalities in fragile X syndrome neurons
Source: Front Neurosci. 2024 Feb 21;18:1348478. doi: 10.3389/fnins.2024.1348478 (PMC10915284; doi:10.3389/fnins.2024.1348478)

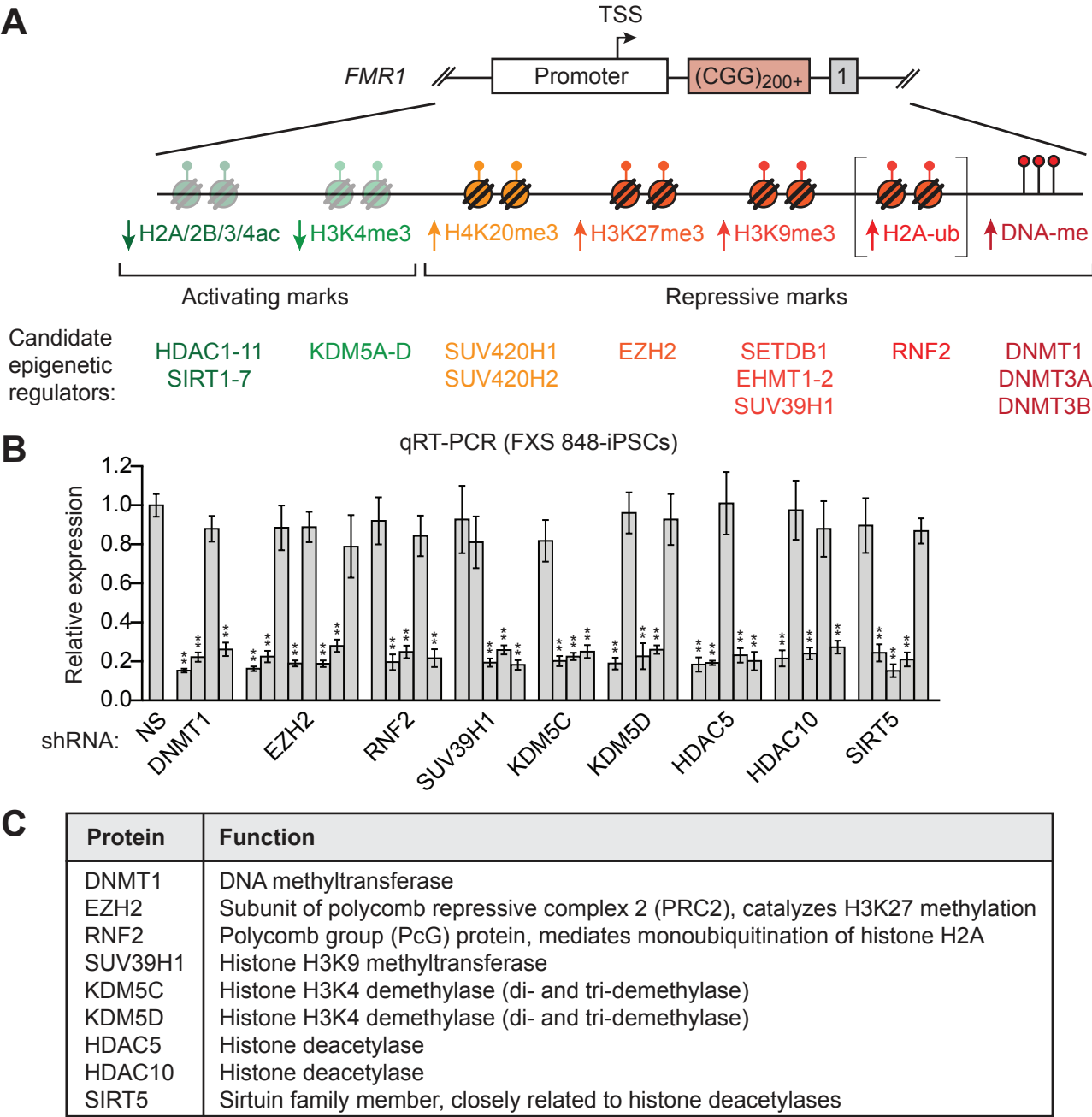

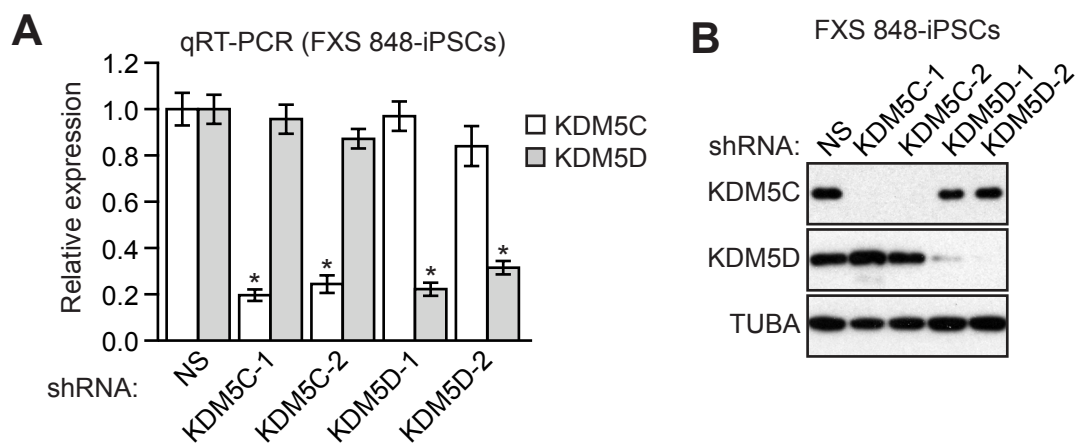

**A**

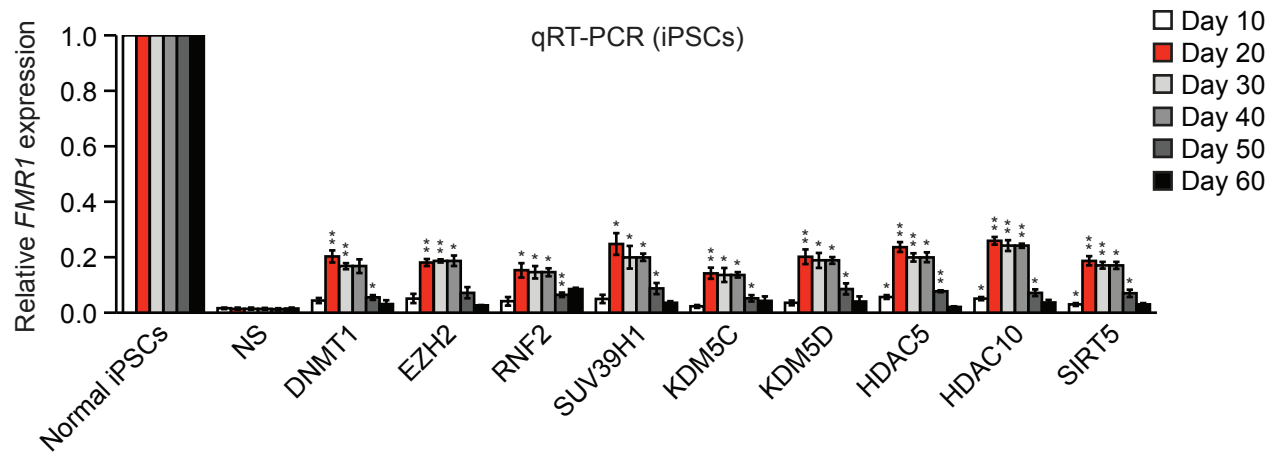

**B**

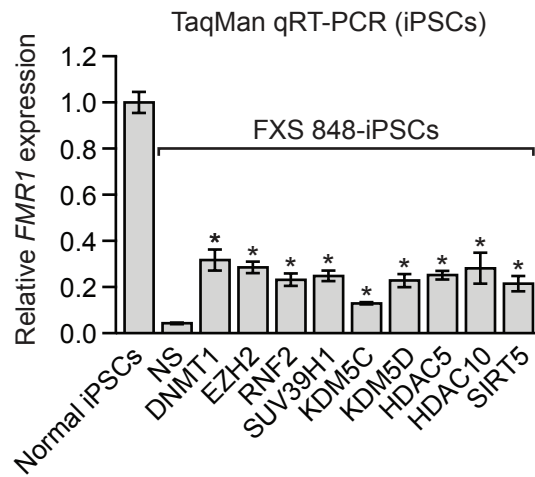

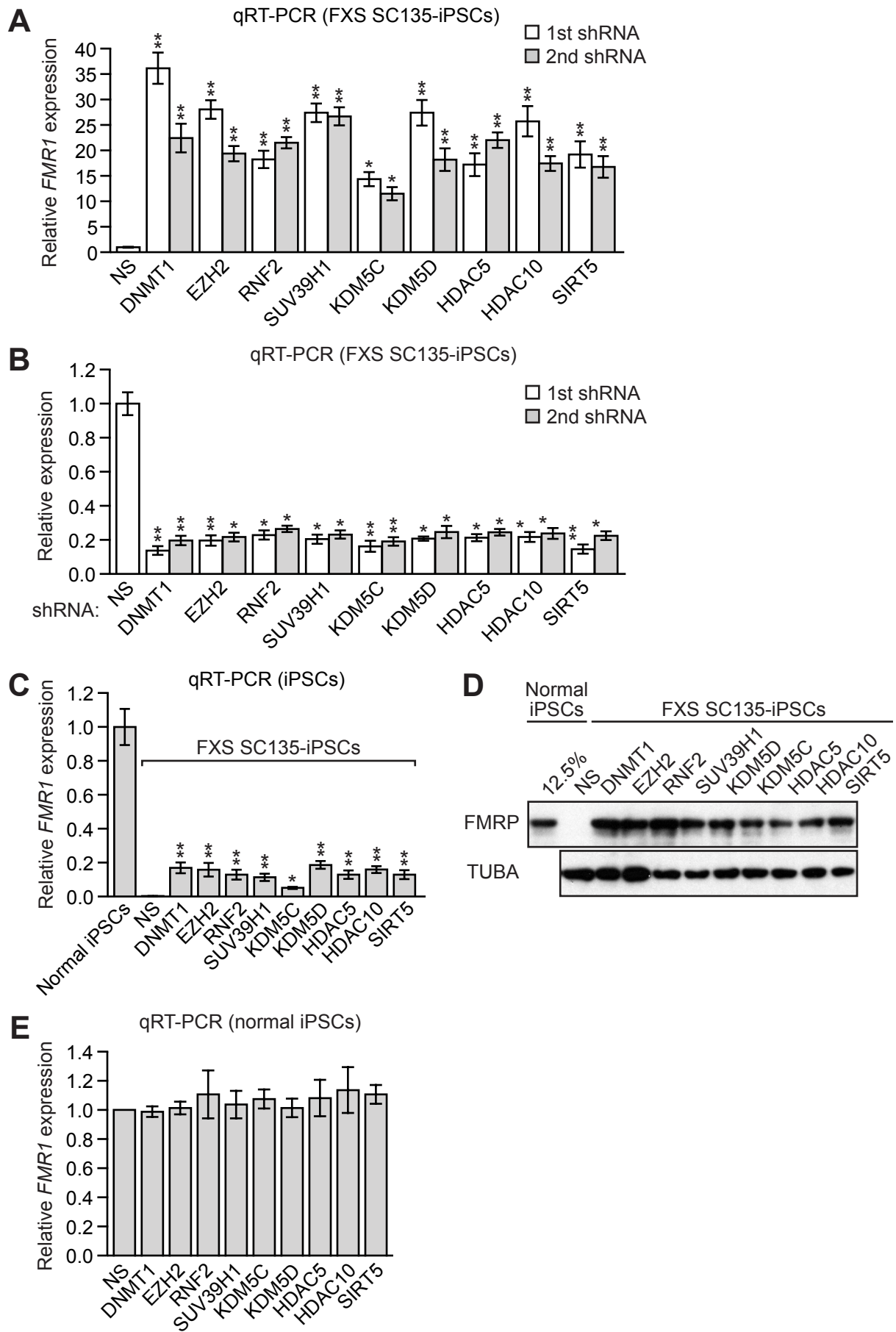

Figure S5

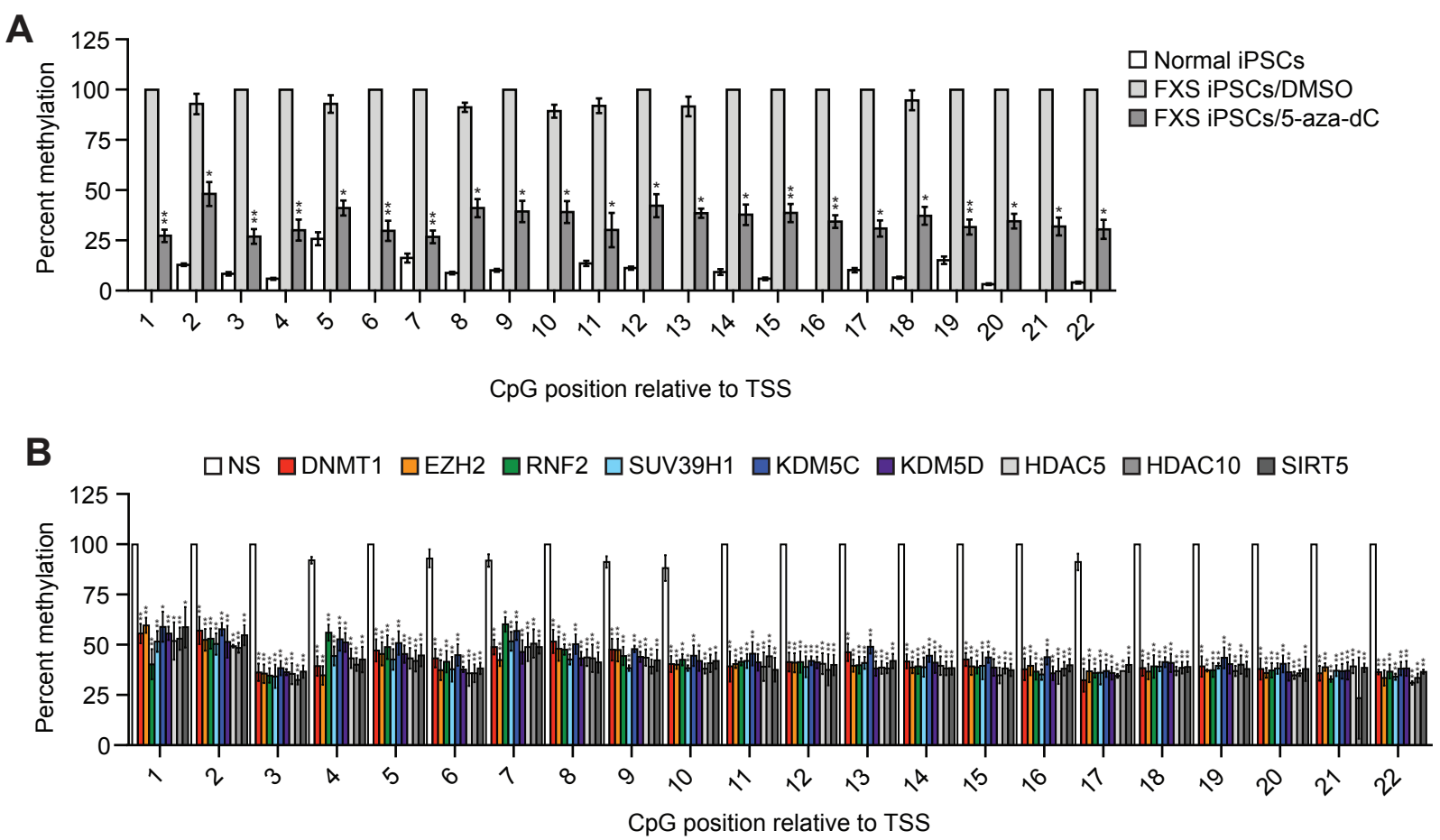

Figure S6

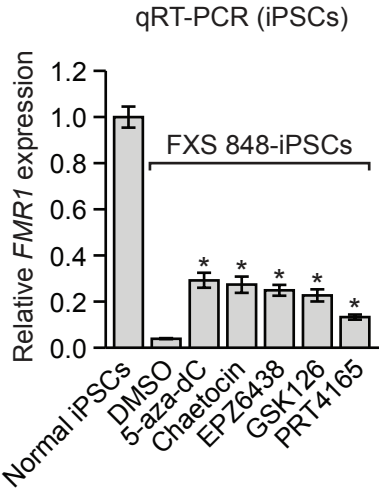

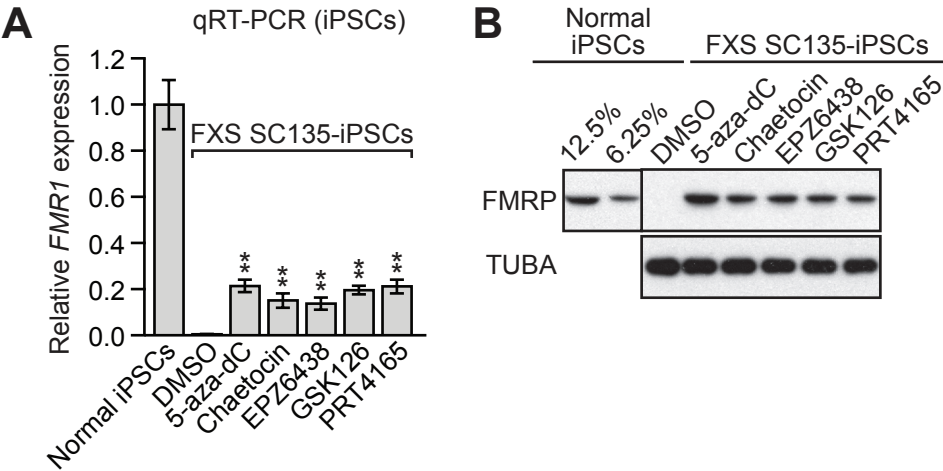

Figure S8

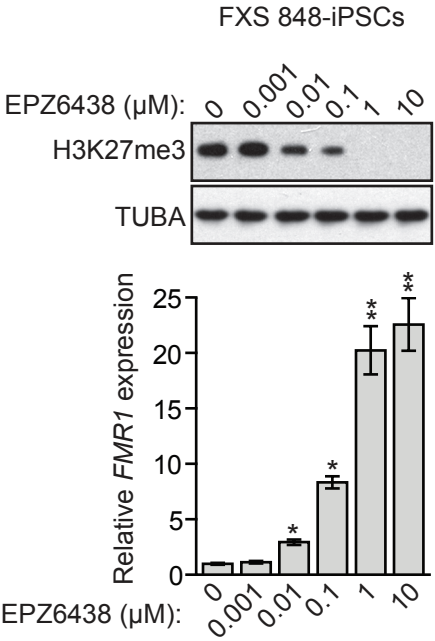

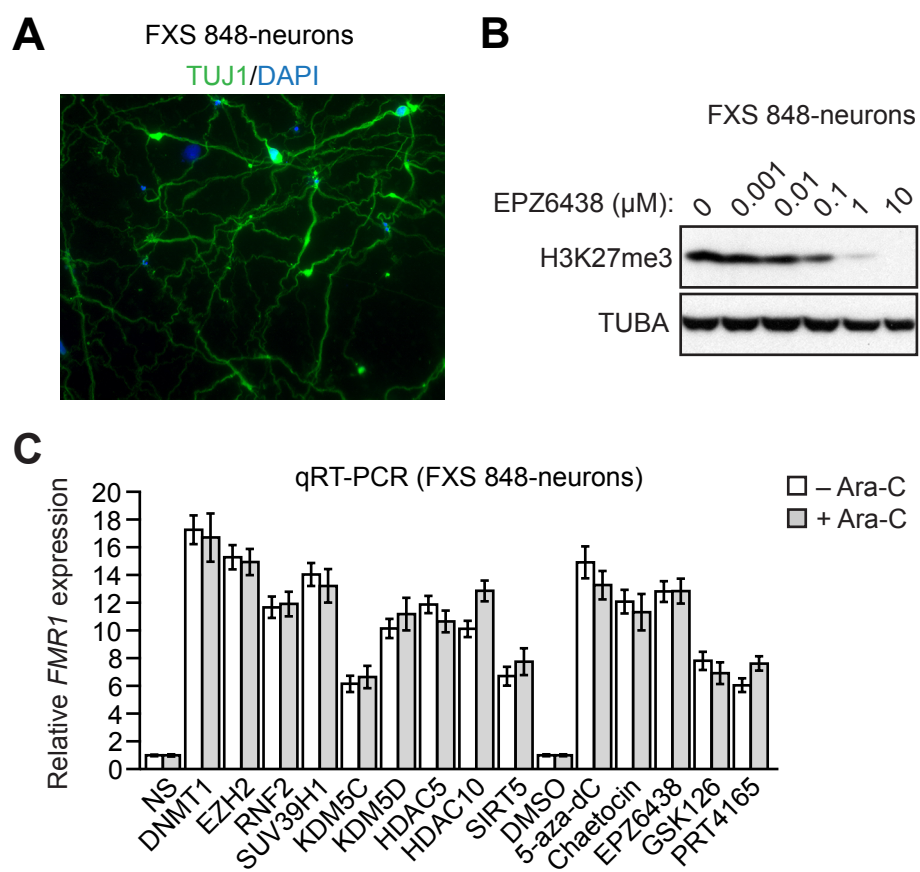

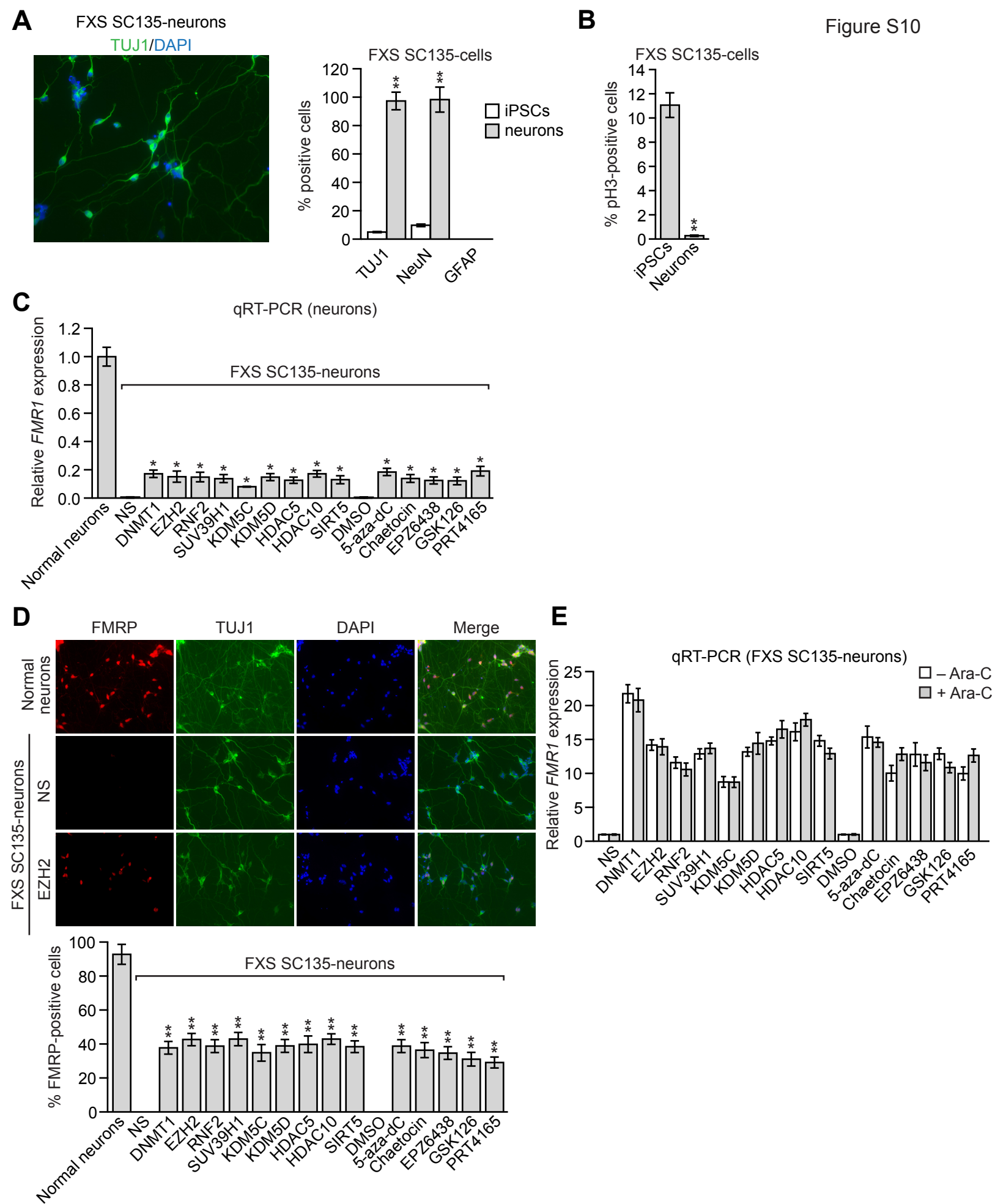

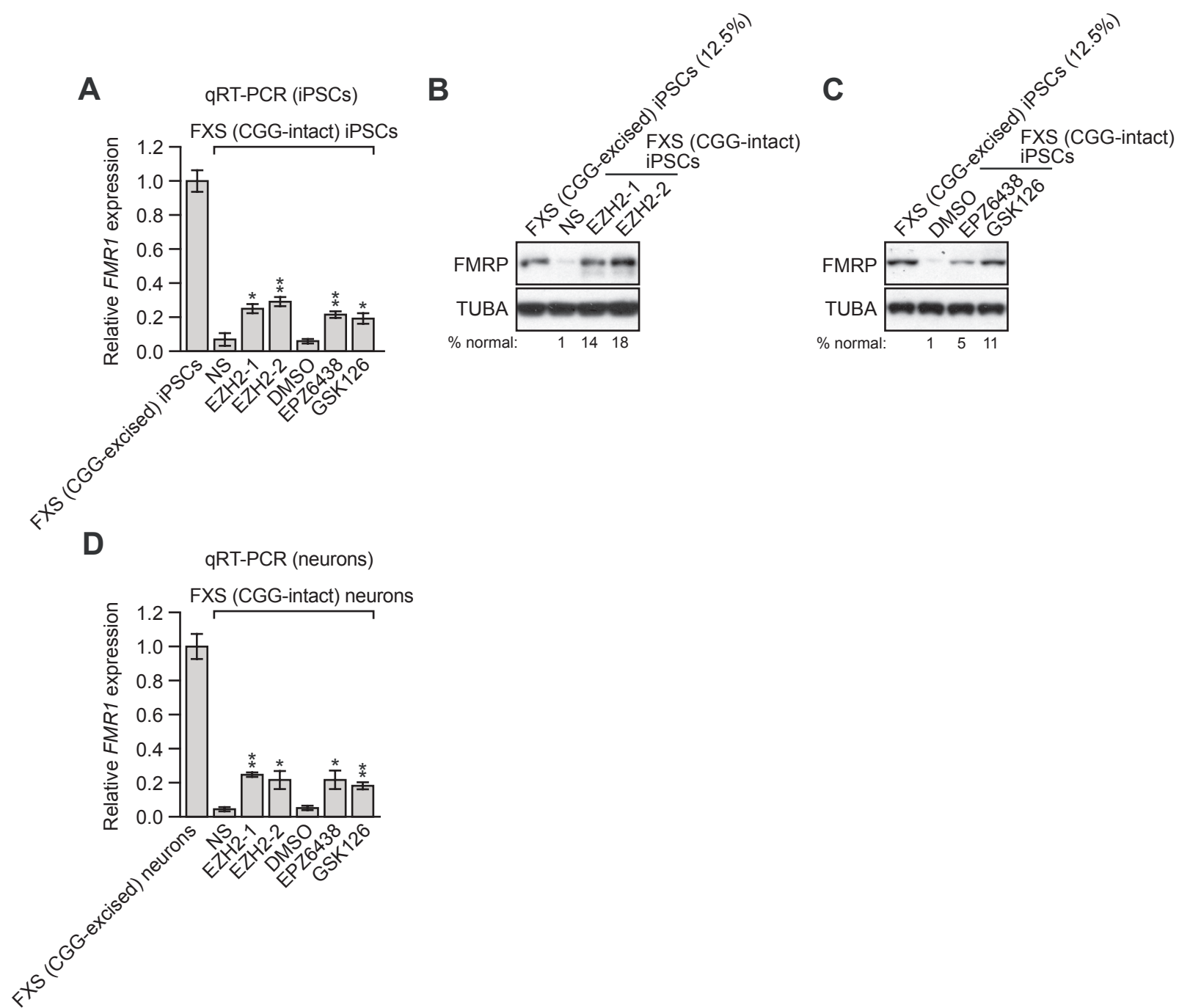

Figure S12

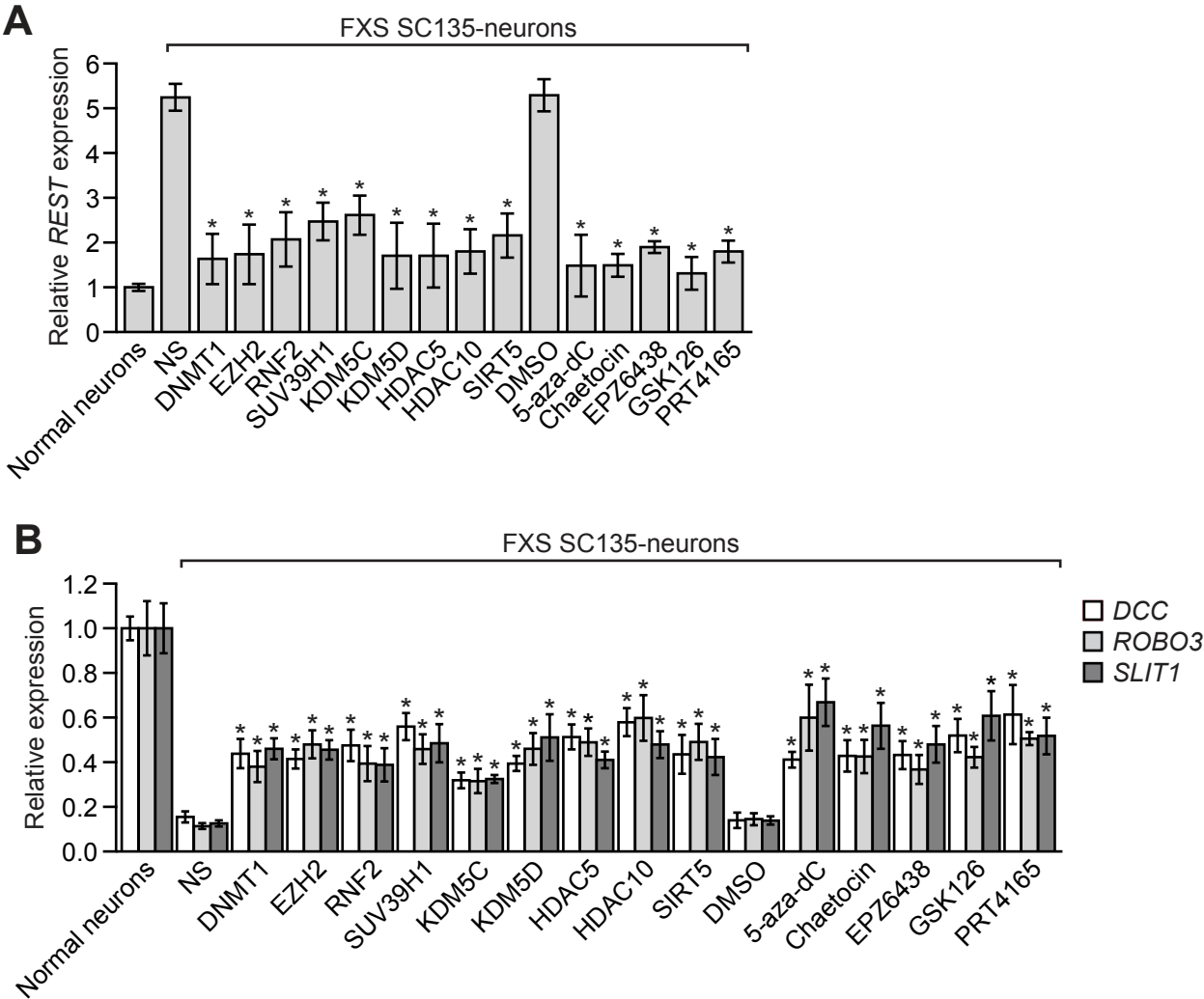

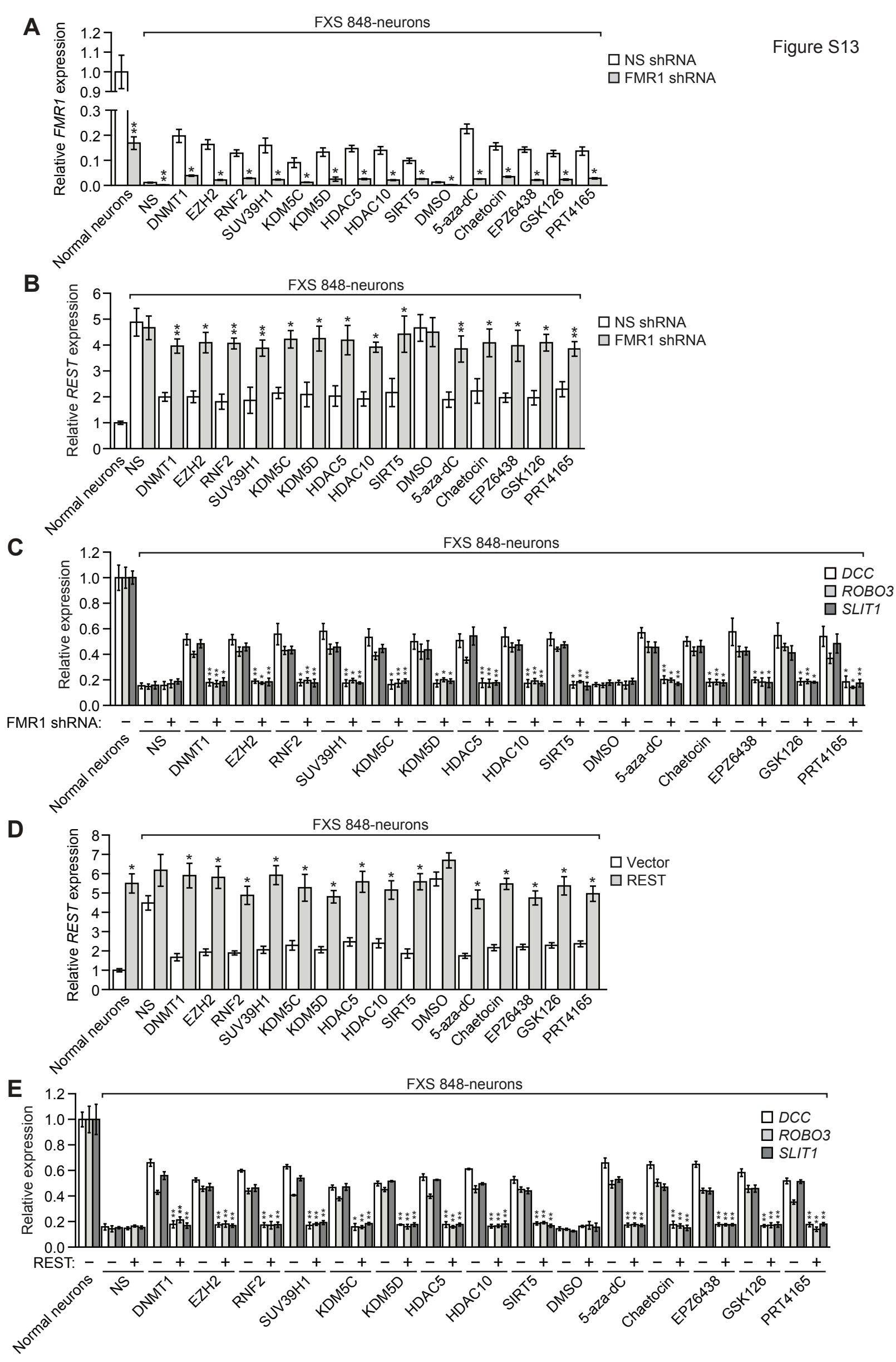

**A**

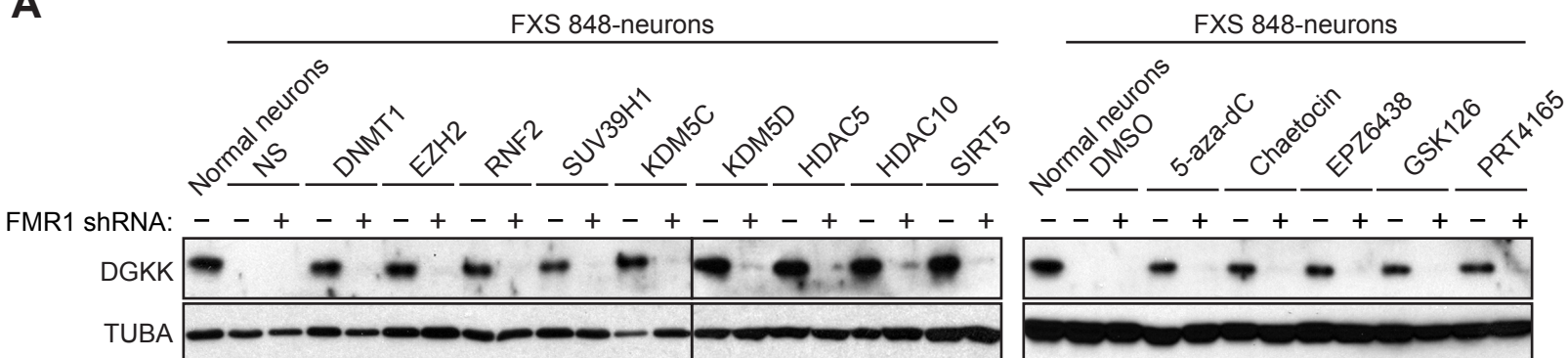

# B

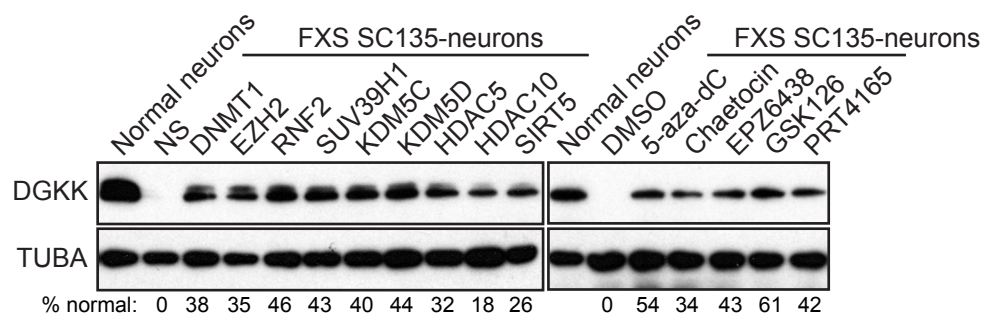

C

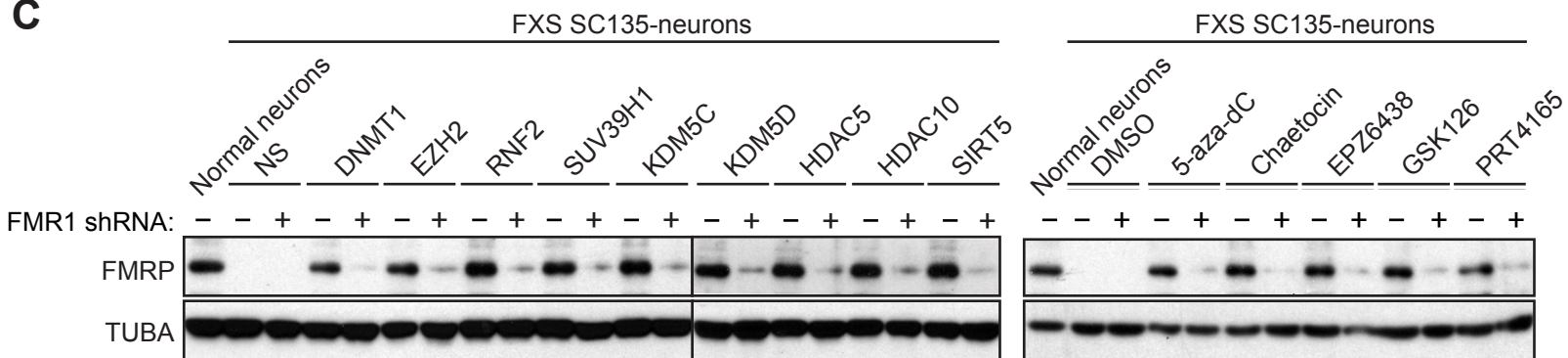

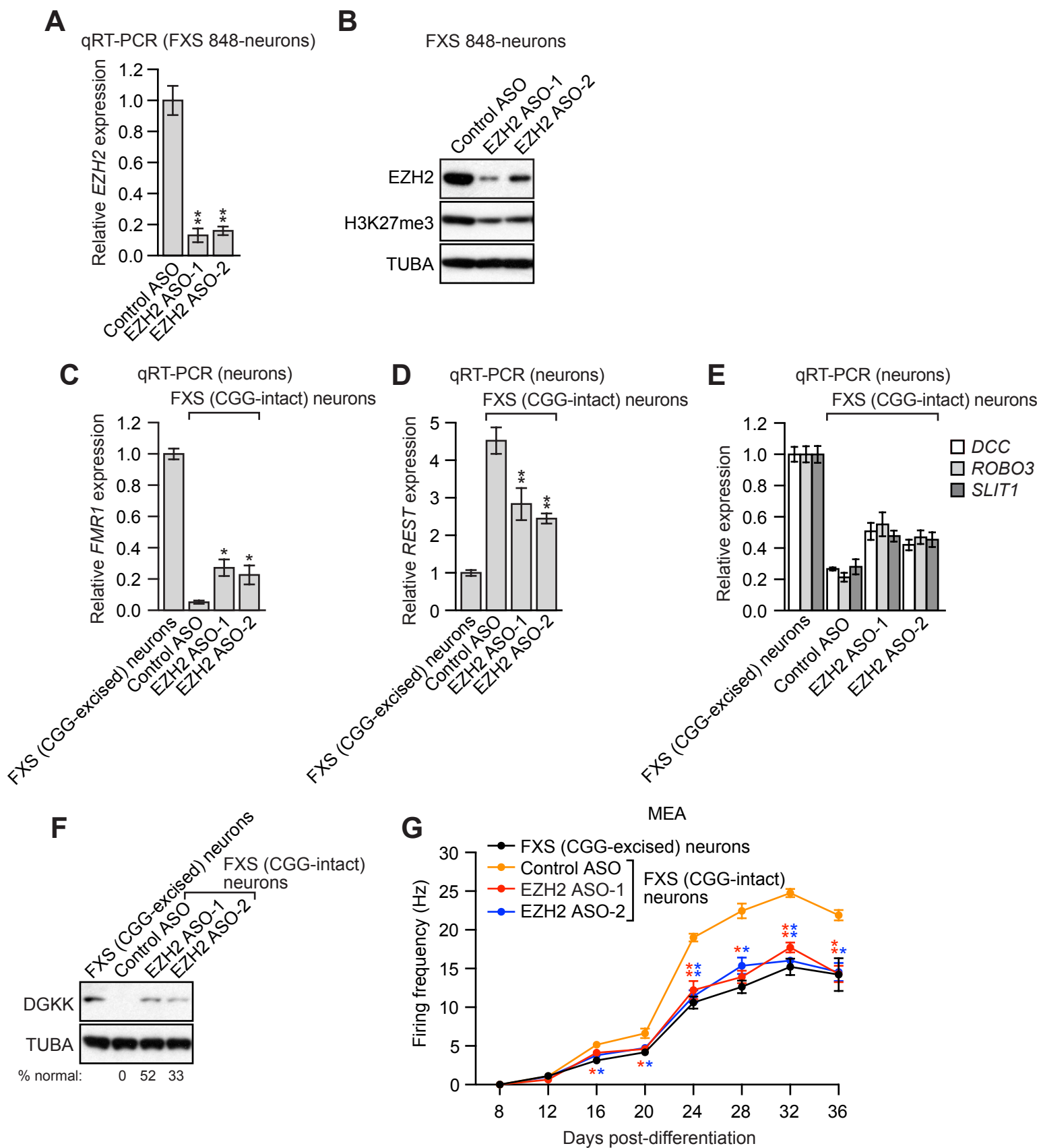

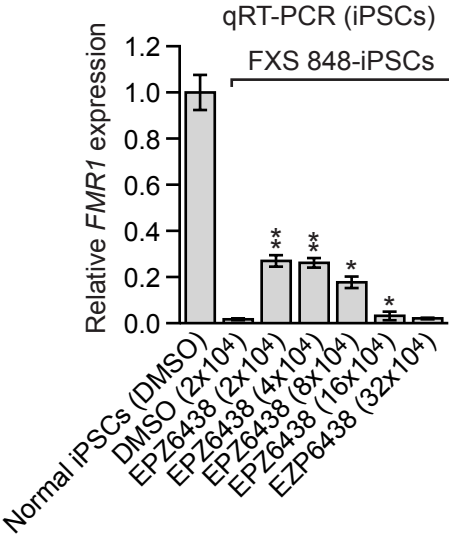

Supplement: Supplementary file 2 [file Data_Sheet_1.PDF]
