# Supplementary material for: EZH2 inhibition reactivates epigenetically silenced FMR1 and normalizes molecular and electrophysiological abnormalities in fragile X syndrome neurons
Source: Front Neurosci. 2024 Feb 21;18:1348478. doi: 10.3389/fnins.2024.1348478 (PMC10915284; doi:10.3389/fnins.2024.1348478)
Supplement: Supplementary file 3 [file Data_Sheet_2.docx]

**SUPPLEMENTARY FIGURE LEGENDS**

**Fig. S1. Identification of epigenetic repressors that mediate silencing of *FMR1* in FXS 848-iPSCs.** **(A)** (Top) Schematic diagram of the promoter and CGG repeat expansion in the 5’ untranslated region of the *FMR1* gene, as well as the known activating and repressive epigenetic marks. TSS, transcription start site. (Bottom) Thirty-three epigenetic repressors that write or erase the known activating and repressive epigenetic marks on the *FMR1* promoter, and were thus analyzed in this study. **(B)** qRT-PCR analysis monitoring knockdown efficiencies of the shRNAs targeting the FMR1-SFs in FXS 848-iPSCs. The results were normalized to that obtained with a control non-silencing (NS) shRNA, which was set to 1. The results show that FMR1-SF shRNAs that failed to upregulate *FMR1* in Fig. 1A also failed to efficiently knock down target gene expression. Data are represented as mean ± SD (n=3 biological replicates). *P<0.05, **P<0.01. **(C)** List of the nine FMR1-SFs and their functions.

**Fig. S2. Confirmation of the specificity of *KDM5C* and *KDM5D* shRNAs.** **(A)** qRT-PCR analysis monitoring expression of *KDM5C* or *KDM5D* in FXS 848-iPSCs expressing an NS shRNA or one of two unrelated *KDM5C* or *KDM5D* shRNAs. Data are represented as mean ± SD (n=3 biological replicates). *P<0.05, **P<0.01. **(B)** Immunoblot analysis monitoring levels of KDM5C or KDM5D in FXS 848-iPSCs expressing an NS shRNA or one of two unrelated *KDM5C* or *KDM5D* shRNAs. α-tubulin (TUBA) was monitored as a loading control.

**Fig. S3. Additional experiments confirming *FMR1* reactivation following knockdown of FMR1-SFs. (A)** Time course of *FMR1* reactivation. qRT-PCR analysis monitoring *FMR1* expression in FXS 848-iPSCs 10-60 days following infection with a lentivirus expressing an FMR1-SF shRNA. The results were normalized to that obtained in normal iPSCs, which was set to 1. The results show that optimal *FMR1* reactivation occurred 20 days following lentiviral transduction. **(B)** Confirmation of *FMR1* reactivation using a TaqMan assay. qRT-PCR analysis using a TaqMan probe monitoring *FMR1* expression in FXS 848-iPSCs 20 days following expression of an FMR1-SF shRNA. The results were normalized to that obtained in normal iPSCs, which was set to 1. Data are represented as mean ± SD (n=3 biological replicates). *P<0.05, **P<0.01.

**Fig. S4. Confirmation of reactivation of epigenetically silenced *FMR1* following knockdown of an FMR1-SF in a second FXS iPSC line, FXS SC135-iPSCs.** **(A)** qRT-PCR analysis monitoring expression of *FMR1* in FXS SC135-iPSCs expressing one of two unrelated shRNAs targeting an FMR1-SF. The results were normalized to that obtained with a control NS shRNA, which was set to 1. **(B)** qRT-PCR analysis monitoring knockdown efficiency of the FMR1-SFs in FXS SC135-iPSCs using two unrelated shRNAs. **(C)** qRT-PCR analysis monitoring *FMR1* expression in FXS SC135-iPSCs expressing an FMR1-SF shRNA. The results were normalized to that obtained in normal iPSCs, which was set to 1. **(D)** Immunoblot analysis showing FMRP protein levels in FXS SC135-iPSCs expressing an FMR1-SF shRNA. The levels of FMRP in normal iPSCs, diluted 8-fold (representing the level of FMRP at 12.5% of normal levels), are shown. **(E)** qRT-PCR analysis monitoring *FMR1* expression in normal iPSCs expressing an FMR1-SF shRNA. The results show that FMR1-SF knockdown in normal iPSCs does not significantly alter *FMR1* expression. Data are represented as mean ± SD (n=3 biological replicates). *P<0.05, **P<0.01. In (E) there was no statistical difference in *FMR1* expression between normal iPSCs expressing an NS or FMR1-SF shRNA.

**Fig. S5. Bisulfite sequencing analysis of the *FMR1* promoter following treatment with 5-aza-dC or an FMR1-SF shRNA. (A)** Quantification of CpG methylation in the *FMR1* promoter in normal iPSCs and FXS 848-iPSCs treated with DMSO or 5-aza-dC. **(B)** Quantification of CpG methylation in the *FMR1* promoter in FXS 848-iPSCs treated with an NS or FMR1-SF shRNA. Multiple independent clones were subjected to bisulfite sequencing, and the percent methylation at each CpG site (numbered relative to the transcription start site (TSS)) was calculated. Data are represented as mean ± SD (n=12-16 biological replicates). *P<0.05, **P<0.01. P value comparisons are between DMSO- and 5-aza-dC-treated FXS iPSCs (A) or between FMR1-SF and NS shRNA-treated iPSCs (B).

**Fig. S6. Confirmation of *FMR1* reactivation levels in FXS 848-iPSCs, following treatment with a small molecule FMR1-SF inhibitor, using a TaqMan assay.** qRT-PCR analysis using a TaqMan probe monitoring *FMR1* expression in FXS 848-iPSCs treated with 5-aza-dC, chaetocin, EPZ6438, GSK126, PRT4165 or, as a control, DMSO. The results were normalized to that obtained in normal iPSCs, which was set to 1. The normal iPSC control was the same as that used in fig. S3B. Data are represented as mean ± SD (n=3 biological replicates). *P<0.05, **P<0.01.

**Fig. S7. Confirmation of reactivation of epigenetically silenced *FMR1* following small molecule inhibition of an FMR1-SF in a second FXS iPSC line, FXS SC135-iPSCs. (A)** qRT-PCR analysis monitoring *FMR1* expression in FXS SC135-iPSCs treated with 5-aza-dC, chaetocin, EPZ6438, GSK126, PRT4165 or, as a control, DMSO. The results were normalized to that obtained in normal iPSCs, which was set to 1. The normal iPSC control used here was the same as that used in fig. S4C. Data are represented as mean ± SD (n=3 biological replicates). *P<0.05, **P<0.01. **(B)** Immunoblot analysis monitoring FMRP levels in FXS SC135-iPSCs treated with 5-aza-dC, chaetocin, EPZ6438, GSK126, PRT4165 or, as a control, DMSO. The levels of FMRP in normal iPSCs, diluted 8-fold or 16-fold (representing the level of FMRP at 12.5% or 6.25%, respectively, of normal levels), are shown. The black vertical line indicates intervening lanes have been spliced out from the same blot.

**Fig. S8. Correlation between loss of EZH2 enzymatic activity and reactivation of epigenetically silenced *FMR1* following treatment with EPZ6483.** (Top) Immunoblot analysis monitoring total H3K27me3 levels in FXS 848-iPSCs treated with increasing concentrations of EPZ6438. (Bottom) qRT-PCR analysis monitoring *FMR1* expression in FXS 848-iPSCs treated with increasing concentrations of EPZ6438. Data are represented as mean ± SD (n=3 biological replicates). *P<0.05, **P<0.01.

**Fig. S9. Additional experiments related to Figure 4.** **(A)** A representative micrograph showing TUJ1-positive neurons derived from FXS 848-iPSCs. TUJ1 staining is shown in green, and DAPI staining in blue. **(B)** Immunoblot analysis monitoring total H3K27me3 levels in FXS 848-neurons treated with increasing concentrations of EPZ6438 for 96 hours. α-tubulin (TUBA) was monitored as a loading control. The results confirm that treatment of post-mitotic neurons with as little as 1 µM EPZ6438 results in a substantial loss of total H3K27me3 levels within 96 hours, the time-course of when *FMR1* reactivation was monitored. **(C)** qRT-PCR analysis monitoring *FMR1* expression in FXS 848-neurons expressing an FMR1-SF shRNA or treated with a small molecule FMR1-SF inhibitor, in the presence or absence of the DNA synthesis inhibitor Ara-C, which kills proliferating cells. Data are represented as mean ± SD (n=3 biological replicates). There was no statistical difference in *FMR1* expression between control and Ara-C-treated cells.

**Fig. S10. Confirmation of reactivation of epigenetically silenced *FMR1* following knockdown or small molecule inhibition of an FMR1-SF in a second FXS neuronal line, FXS SC135-neurons. (A)** (Left) A representative micrograph showing TUJ1-positive neurons derived from FXS SC135-iPSCs. TUJ1 staining is shown in green, and DAPI staining in blue. (Right) Percentage of TUJ1-, NeuN- and GFAP-positive cells. Data are represented as mean ± SD (n=3 biological replicates with at least 300 cells analyzed per sample). **(B)** Percentage of phosphorylated H3-positive cells in FXS SC135-iPSCs and FXS SC135-neurons. Data are represented as mean ± SD (n=3 biological replicates with at least 300 cells analyzed per sample). The results confirm that FXS SC135-neurons are post-mitotic. **(C)** qRT-PCR analysis monitoring *FMR1* expression in FXS SC135-neurons expressing an FMR1-SF shRNA or treated with a small molecule FMR1-SF inhibitor. The results were normalized to that obtained in normal neurons, which was set to 1. Data are represented as mean ± SD (n=3 biological replicates). **(D)** (Top) ICC monitoring FMRP levels in normal neurons and FXS SC135-neurons expressing a NS or EZH2 shRNA. (Bottom) Quantification of the percentage of FMRP-positive cells in FXS SC135-neurons expressing an shRNA or small molecule FMR1-SF inhibitor. Data are represented as mean ± SD (n=3 biological replicates with at least 300 cells analyzed per sample). **(E)** qRT-PCR analysis monitoring *FMR1* expression in FXS SC135-neurons expressing an FMR1-SF shRNA or treated with a small molecule FMR1-SF inhibitor, and treated in the presence or absence of Ara-C. Data are represented as mean ± SD (n=3 biological replicates). *P<0.05, **P<0.01. In (E) there was no statistical difference in *FMR1* expression between control and Ara-C-treated cells, demonstrating that the observed *FMR1* reactivation in FXS SC135-neurons is from post-mitotic cells.

**Fig. S11. Confirmation of reactivation of epigenetically silenced *FMR1* following knockdown or small molecule inhibition of EZH2 in an isogenic pair of FXS cell lines. (A)** qRT-PCR analysis monitoring *FMR1* expression in FXS (CGG-intact) iPSCs expressing an NS or EZH2 shRNA or treated with a small molecule EZH2 inhibitor, EPZ6438 or GSK126. The results were normalized to that obtained in FXS (CGG-excised) iPSCs, which was set to 1. **(B,C)** Immunoblot analysis monitoring FMRP levels in FXS (CGG-intact) iPSCs expressing an NS or EZH2 shRNA (B) or treated with DMSO, EPZ6438 or GSK126 (C). The levels of FMRP in FXS (CGG-excised) iPSCs, diluted 8-fold (representing the level of FMRP at 12.5% of normal levels), are shown. **(D)** qRT-PCR analysis monitoring *FMR1* expression in FXS (CGG-intact) neurons expressing an NS or EZH2 shRNA or treated with a small molecule EZH2 inhibitor, EPZ6438 or GSK126. The results were normalized to that obtained in FXS (CGG-excised) neurons, which was set to 1. Data are represented as mean ± SD (n= 3 biological replicates). *P<0.05, **P<0.01.

**Fig. S12. Confirmation of normalized expression of *REST* and REST target genes following knockdown or small molecule inhibition of an FMR1-SF in a second FXS neuronal line, FXS SC135-neurons. (A,B)** qRT-PCR analysis monitoring expression of *REST* (A) or *DCC, ROBO3* and *SLIT1* (B) in FXS SC135-neurons expressing an FMR1-SF shRNA or treated with a small molecule FMR1-SF inhibitor. The expression of *FMR1* in FXS SC135-neurons is shown relative to that in normal neurons, which was set to 1. Data are represented as mean ± SD (n= 3 biological replicates). *P<0.05, **P<0.01.

**Fig. S13. Normalized expression of *REST*, *DCC*, *ROBO3* and *SLIT1* is a direct effect of *FMR1* reactivation. (A)** qRT-PCR analysis monitoring *FMR1* expression in FXS 848-neurons expressing an NS or *FMR1* shRNA, and expressing an FMR1-SF shRNA or treated with a small molecule FMR1-SF inhibitor. The results were normalized to that obtained in normal neurons, which was set to 1. The results confirm efficient *FMR1* knockdown upon treatment with an *FMR1* shRNA in FXS 848-neurons treated with an FMR1-SF shRNA or small molecule FMR1-SF inhibitor. **(B,C)** qRT-PCR analysis monitoring expression of *REST* (B) or *DCC*, *ROBO3* and *SLIT1* (C) in FXS 848-neurons expressing an NS or *FMR1* shRNA, and expressing an FMR1-SF shRNA or treated with a small molecule FMR1-SF inhibitor. **(D)** qRT-PCR analysis monitoring *REST* expression in FXS 848-neurons expressing an FMR1-SF shRNA and co-expressing empty vector or REST. The results were normalized to that obtained in normal neurons expressing empty vector, which was set to 1. The results confirm that *REST* is overexpressed in neurons expressing a *REST* expression vector. **(E)** qRT-PCR analysis monitoring expression of *DCC*, *ROBO3* and *SLIT1* in FXS 848-neurons expressing empty vector or *REST*, and expressing an FMR1-SF shRNA or treated with a small molecule FMR1-SF inhibitor. The results show that ectopic expression of *REST* restores normal expression levels of REST target genes in FXS 848-neurons treated with an FMR1-SF shRNA or small molecule FMR1-SF inhibitor. Data are represented as mean ± SD (n=3 biological replicates). *P<0.05, **P<0.01.

**Fig. S14. *FMR1* knockdown restores normal DGKK levels in FXS neurons treated with an FMR1-SF shRNA or small molecule FMR1-SF inhibitor.** **(A)** Immunoblot analysis showing DGKK levels in FXS 848-neurons expressing an NS or *FMR1* shRNA, and expressing an FMR1-SF shRNA or treated with a small molecule FMR1-SF inhibitor. **(B)** Immunoblot analysis showing DGKK levels in FXS SC135-neurons expressing an FMR1-SF shRNA or treated with a small molecule FMR1-SF inhibitor. DGKK levels in normal neurons are shown. The DGKK signal was quantified and normalized to that obtained in normal neurons, which was set to 100%. **(C)** Immunoblot analysis showing DGKK levels in FXS SC135-neurons expressing an NS or *FMR1* shRNA, and expressing an FMR1-SF shRNA or treated with a small molecule FMR1-SF inhibitor.

**Fig. S15. Confirmation of EZH2 ASO-mediated *FMR1* reactivation and normalization of molecular and electrophysiological abnormalities in an isogenic pair of FXS neuron lines. (A)** qRT-PCR analysis monitoring *EZH2* expression in FXS 848-neurons treated with a control or EZH2 ASO. **(B)** Immunoblot monitoring levels of H3K27me3 in FXS 848-neurons expressing an EZH2 ASO or a control ASO. The results confirm that EZH2 ASOs reduce the levels of H3K27me3 in human cells. **(C-E)** qRT-PCR analysis monitoring *FMR1* (C), *REST* (D) or *DCC, ROBO3* and *SLIT1* (E) in FXS (CGG-intact)-neurons treated with a control or EZH2 ASO. The results were normalized to that obtained in FXS (CGG-excised)-neurons, which was set to 1. (**F**) Immunoblot analysis showing DGKK levels in cultured FXS (CGG-intact)-neurons treated with a control or EZH2 ASO. (**G**) MEA showing firing frequency of cultured FXS (CGG-intact)-neurons treated with a control or EZH2 ASO. The firing frequency of FXS (CGG-excised) neurons is shown. Data are represented as mean ± SD (n=3 biological replicates). *P<0.05, **P<0.01.

**Fig. S16. The level of *FMR1* reactivation is cell density dependent.** FXS 848-iPSCs were seeded in 12-well plates at varying densities (2-32x10^4^ cells per well) and 24 hours later treated with 1 µM EPZ6438, or as a control DMSO, for 72 hours. The results were normalized to that obtained in normal iPSCs treated with DMSO, which was set to 1. Data are represented as mean ± SD (n=3 biological replicates). Data are represented as mean ± SD (n=3 biological replicates). *P<0.05, **P<0.01.

**Table S1.** List of 33 epigenetic regulators and corresponding shRNAs used in the candidate-based shRNA screen. Positive shRNAs used this study are indicated.

See the accompanying Excel file.

**Table S2.** List of primers used in this study.

| **Gene** | **Forward primer sequence (5’🡪3’)** | **Reverse primer sequence (5’🡪3’)** |
| --- | --- | --- |
| **qRT-PCR** |  |  |
| *hRPL41* | CATTAAATAGCCGTAGACGGAACTT | GCGCAGAGGTTTCCAAAAAA |
| *DCC* | AGTTGCCAAGACCTCACACC | TCCCTGCTTCCTCAGTGTCT |
| *DNMT1* | GCCTGAGAACACCCACAAGT | ACAGCCTTGAAGTCCACCAC |
| *EZH2* | TGATAGGGAAGCAGGGACTG | CCGAGAATTTGCTTCAGAGG |
| *FMR1* | GGTGAGGATTGAGGCTGAAA | GCATTAGGTCCAACCCTTGA |
| *HDAC5* | TCCTCCTCCTTCCTCTTGGT | TGTGAGGAGTGTGAGGCAAG |
| *HDAC10* | CACATGCCAAGCAGAAACAC | CTGGGGTCATCCTCAAAGAG |
| *KDM5C* | CTCAGTCCGGGGTGGTACT | CCAAGCCCTAGGAAACCTTC |
| *KDM5D* | AATACAAGCCCCACAGCATC | AGGCTCTGGATCAGGCTGTA |
| *REST* | CCTTTCCAAAGCTGAACTGG | ACCGACCAGGTAATCACAGC |
| *RNF2* | ATTGGCCATCACTACCAACC | CCACTTGTGGTGAGCCTTTT |
| *ROBO3* | GCCAATGCCTGAGAGAAGTC | CCATAGGAAGGTGTGGGAGA |
| *SLIT1* | GTCAGCACTCACGTTGCCTA | GGGGACAAGATGTGGAGAGA |
| *SIRT5* | CCCTTGCCTGTCATGAAAAT | ACCATTTCTCCTCTGCGTGT |
| *SUV39H1* | CTTCTGCCTGGAGATTGAGG | TAGGCACTCTGGCCTCTGAT |
| **ChIP** |  |  |
| *APRT* | GCCTTGACTCGCACTTTTGT | TAGGCGCCATCGATTTTAAG |
| *FMR1* | ACAGTGGAATGTAAAGGGTTG | GTGTTAAGCACTTGAGGTTCAT |
| *GCLC* | ACCGCCTCCCCGTGACTCAG | CAGCAGCAGCAGCCCAGAGG |
